# Metabolic Effects Associated with ICS in Patients with COPD and Comorbid Type 2 Diabetes: A Historical Matched Cohort Study

**DOI:** 10.1371/journal.pone.0162903

**Published:** 2016-09-22

**Authors:** David B. Price, Richard Russell, Rafael Mares, Anne Burden, Derek Skinner, Helga Mikkelsen, Cherlyn Ding, Richard Brice, Niels H. Chavannes, Janwillem W. H. Kocks, Jeffrey W. Stephens, John Haughney

**Affiliations:** 1 Academic Primary Care, University of Aberdeen, Aberdeen, United Kingdom; 2 Observational and Pragmatic Research Institute, Singapore, Singapore; 3 Nuffield Department of Medicine, University of Oxford, Oxford, United Kingdom; 4 Research in Real Life Ltd, Cambridge, United Kingdom; 5 Cambridge Research Support, Cambridge, United Kingdom; 6 Optimum Patient Care, Cambridge, United Kingdom; 7 Whitstable Medical Practice, Whitstable, Canterbury, United Kingdom; 8 Department of Public Health and Primary Care, Leiden University Medical Center, Leiden, the Netherlands; 9 Department of General Practice and GRIAC Research Institute, University Medical Center Groningen, Groningen, the Netherlands; 10 Diabetes Research Group, Institute of Life Sciences, Swansea University, Swansea, United Kingdom; Brown University, UNITED STATES

## Abstract

**Background:**

Management guidelines for chronic obstructive pulmonary disease (COPD) recommend that inhaled corticosteroids (ICS) are prescribed to patients with the most severe symptoms. However, these guidelines have not been widely implemented by physicians, leading to widespread use of ICS in patients with mild-to-moderate COPD. Of particular concern is the potential risk of worsening diabetic control associated with ICS use.

Here we investigate whether ICS therapy in patients with COPD and comorbid type 2 diabetes mellitus (T2DM) has a negative impact on diabetic control, and whether these negative effects are dose-dependent.

**Methods and Findings:**

This was a historical matched cohort study utilising primary care medical record data from two large UK databases. We selected patients aged ≥40 years with COPD and T2DM, prescribed ICS (n = 1360) or non-ICS therapy (n = 2642) between 2008 and 2012. The primary endpoint was change in HbA_1c_ between the baseline and outcome periods. After 1:1 matching, each cohort consisted of 682 patients. Over the 12–18-month outcome period, patients prescribed ICS had significantly greater increases in HbA_1c_ values compared with those prescribed non-ICS therapies; adjusted difference 0.16% (95% confidence interval [CI]: 0.05–0.27%) in all COPD patients, and 0.25% (95% CI: 0.10–0.40%) in mild-to-moderate COPD patients. Patients in the ICS cohort also had significantly more diabetes-related general practice visits per year and received more frequent glucose strip prescriptions, compared with those prescribed non-ICS therapies. Patients prescribed higher cumulative doses of ICS (>250 mg) had greater odds of increased HbA_1c_ and/or receiving additional antidiabetic medication, and increased odds of being above the Quality and Outcomes Framework (QOF) target for HbA_1c_ levels, compared with those prescribed lower cumulative doses (≤125 mg).

**Conclusion:**

For patients with COPD and comorbid T2DM, ICS therapy may have a negative impact on diabetes control. Patients prescribed higher cumulative doses of ICS may be at greater risk of diabetes progression.

**Trial Registration:**

ENCePP ENCEPP/SDPP/6804

## Introduction

Chronic obstructive pulmonary disease (COPD) is a common and underdiagnosed medical condition that is projected to become the third leading cause of death worldwide by 2020.[1, 2] Current UK and international guidelines on COPD management recommend initial treatment with long-acting muscarinic antagonists (LAMA) or long-acting β_2_-agonists (LABA), with high-dose inhaled corticosteroids (ICS) reserved for patients with more severe disease. Bronchodilators can be prescribed as monotherapy, in combination with each other, or with an additional drug (eg LABA/LAMA or ICS/LABA).[3, 4] There is evidence to suggest that ICS/LABA therapy reduces exacerbations and increases quality of life in appropriate patients.[5] However, an increasing body of evidence suggests a link between the prescription of high doses of ICS and the risk of comorbidities, such as cataracts, osteoporosis and pneumonia[6–10]—events clearly associated with systemic/oral corticosteroids. Therapy with ICS has specifically been associated with an increased serum glucose concentration, which may be particularly important for patients with COPD in whom co-morbid type 2 diabetes mellitus (T2DM) is more prevalent than in the general population.[11–13] The potential risk of reduced glycaemic control is of further concern for these patients due to their high obesity rates, and because the licensed doses of ICS are high compared with those prescribed to patients with asthma.[14, 15]

International guidelines recommend that ICS treatment should be reserved for patients with COPD who have severe airflow limitation and/or a high risk of exacerbations, ie groups C and D of the Global Initiative for Chronic Obstructive Lung Disease (GOLD) staging.[3, 4] This is in agreement with current UK license specifications.[5] Contrary to guidelines, however, ICS are frequently prescribed for patients in GOLD groups A and B who have mild or moderate spirometrical COPD, fewer symptoms, and a lower risk of exacerbations than those in groups C and D.[16–18] This means that more patients could be exposed to the potential risks of ICS treatment than necessary. The reasons for these prescribing patterns are likely to be complex and may include diagnostic uncertainty, therapeutic confusion with other respiratory disease management guidelines, or co-existing asthma and COPD.[16–18]

A large study by Suissa et al.[6] based on prescription claims for a broad group of respiratory patients, most of them with a likely COPD diagnosis, suggested that ICS use might be associated with increased diabetes rates. However, there is a lack of real-life biochemical data on the potential adverse effects of ICS treatment on comorbidities in patients with confirmed COPD diagnoses. Further information is needed on the specific effects of ICS therapy on glycaemic control and progression to insulin in patients with COPD and comorbid T2DM. The aim of this historical matched cohort study was to investigate whether ICS therapy in a UK patient population with COPD and comorbid T2DM has a negative impact on diabetes control. Specifically, we assessed whether ICS use is associated with an increase in glycated haemoglobin (HbA_1c_) levels in these patients. In addition, we assessed whether ICS use influences other indicators of diabetes control and the risk of diabetes progression, especially in patients who should not be prescribed ICS according to COPD management guidelines, and whether these potential effects of ICS use are dose-dependent.

## Methods

### Study design

This historical, matched cohort study consisted of a baseline period for patient characterisation, during 2008–2012, followed by an outcome period during which the study endpoints were evaluated. The baseline and outcome periods were separated by the index date, defined as either a first prescription of an ICS (ICS cohort), or a first/additional prescription of non-ICS inhaled respiratory therapy (non-ICS cohort). To capture all valid HbA_1c_ measurements before and after the index date, the baseline and outcome periods covered between 12 and 18 months each.

The study protocol (included in supporting information, as S1 Protocol) was designed prior to data extraction by an independent steering committee and registered with the European Network of Centres for Pharmacoepidemiology and Pharmacovigilance (ENCePP registration number ENCEPP/SDPP/6804).

### Data Source

To maximise the number of patients available, data were extracted from two large UK primary care databases and combined into a single dataset. The Clinical Practice Research Datalink (CPRD) contains de-identified longitudinal data from more than 680 subscribing practices, is well validated and frequently used for medical and health research.[19] The Optimum Patient Care Research Database (OPCRD) is a quality-controlled and respiratory-focused research database containing anonymous, longitudinal data for over two million patients from over 500 UK practices. The database has received a favourable opinion from the Health Research Authority of the UK NHS for clinical research use (REC reference: 15/EM/0150).[20]

### Patients

Eligible patients (≥40 years old at the index date) had a spirometry-test validated Quality and Outcomes Framework (QOF) coded diagnosis of COPD at any time (ratio of forced expiratory volume in 1 second to forced vital capacity [FEV_1_/FVC] <0.7), and a QOF-coded diagnosis of T2DM before the index date. The code lists we used for COPD and T2DM can be found in Supplementary methods (S1 File). The QOF is the UK National Health Service pay-for-performance scheme that provides an incentive for practices to deliver better patient care and includes the creation of high-quality electronic disease registers[21, 22], and is known to have improved diabetes care.[23] Patients had to have at least 1 full year of data available during the baseline period, including HbA_1c_ data available at least once within the baseline period, as well as at least once between 20 days and 18 months in the outcome period. The 20-day limit was chosen on the basis that HbA_1c_ levels have been reported to plateau 20 days after intensification of glucose lowering therapy and remain comparable for a further 100 days,[24, 25] while the 18 month limit was designed to capture all valid HbA_1c_ readings, assuming HbA_1c_ values were recorded at least every 15 months as per QOF diabetes indicators specified for the study period.[22] For the ICS cohort, patients were included if they had a first prescription for ICS between 2008 and 2012, with a medication possession ratio for ICS of ≥50% in the outcome period.[22] For the non-ICS cohort, patients were included if they had a first or additional prescription for non-ICS therapy between 2008 and 2012, and no ICS therapy prior to an HbA_1c_ reading in the outcome period. Exclusion criteria included any record of type 1 diabetes and any prescriptions for maintenance oral corticosteroids in the year before the index date.

### Study endpoints

Primary and secondary outcomes of the study are outlined in Table 1. The primary outcome measure was a change in HbA_1c_ value, observed over a period of 12–18 months. The change was defined as the difference between baseline and outcome HbA_1c_ values. The baseline HbA_1c_ referred to the most recent value prior to the index date, and the outcome HbA_1c_ referred to the last value in the outcome period. Secondary outcomes focused on other measures of diabetes control, defined as increase in HbA_1c_ and/or addition of antidiabetic drugs, number of patients off the HbA_1c_ QOF target, diabetes-related GP visits, hospitalisations, glucose strip prescriptions, and progression to insulin (Table 1). The time to progression to insulin, and the effects of the cumulative dose of ICS on diabetes control, were also investigated.

**Table 1:** Study endpoints and matching criteria.

**Study endpoints**	
• ***Primary endpoint*** ○ Change in HbA_1c_ from baseline to outcome period	
• ***Secondary endpoints*** ○ Increase in HbA_1c_* and/or the addition of antidiabetic medication from baseline to outcome period ○ Number of patients off the QOF target for HbA_1c_ (HbA_1c_ >7.5%) ○ Change in diabetes-related GP visits, hospitalisations, and glucose strip prescriptions^†^ ○ Progression of ongoing diabetes treatment to insulin by time or dose	
**Matching criteria**	**Categories**
Sex	Male / female
Age (±5 years at index date^‡^)	NA
Smoking status at index date	Non-smoker / current smoker / ex-smoker
BMI at index date	Underweight / normal weight / overweight / obese
mMRC score[4] at index date	Missing / 0–1 / ≥2
Exacerbations^§^ during baseline	0–1 / ≥2
FEV_1_, % predicted at index date	Missing / <50% / ≥50%
HbA_1c_^¶^ ±1 DCCT (%) during baseline	NA
Antidiabetic medication during baseline	None / non-insulin / insulin

*Increase in HbA_1c_ was defined as a change of ≥0.5% from baseline to outcome period. Antidiabetic medication during baseline was categorised as 1, 2 or ≥3 of non-insulin or insulin, and any progression from baseline to outcome within these categories was recorded as addition of an antidiabetic drug.

^†^GP visits: change in annual rate from baseline to outcome period; hospitalisations: increase from baseline to outcome period; glucose strip prescriptions: increase (>0.5 per year) from baseline to outcome period.

^‡^The index date was defined as the date of first ICS prescription or first/additional non-ICS prescription.

^§^Defined as the number of acute oral corticosteroid courses and antibiotic prescriptions for a lower respiratory tract infection.

^¶^Most recent value within 18 months prior to the index date.

BMI = body mass index; DCCT = Diabetes Control and Complications Trial units; FEV_1_ = forced expiratory volume in 1 second; GP = general practice; HbA_1c_ = glycated haemoglobin; mMRC = modified Medical Research Council; NA = not applicable; QOF = Quality and Outcomes Framework.

### Statistical analysis

The study was powered on the primary outcome, change in HbA_1c_ value. Based on a previous study[26] and unpublished data, detecting a mean HbA_1c_ change of 0.25% with 90% power to reject the null hypothesis, (no difference in change and using a two-sided t-test with a 5% significance level), assuming that the common standard deviation is 1.1, required 408 patients in each cohort. Records from the OPCRD and CPRD databases were combined into a single dataset. Patient selection was then carried out according to inclusion/exclusion criteria, resulting in 1360 and 2642 patients in the ICS and non-ICS cohorts, respectively (S1 Fig, S1 File).

A baseline analysis of unmatched patients revealed significant differences between the ICS and non-ICS cohorts (Table 2). As might be expected, patients in the ICS cohort generally had more severe COPD than those in the non-ICS cohort with higher proportions having a modified British Medical Research Council (mMRC) score ≥2,[4] or a %predicted FEV_1_ <50. Higher percentages of patients in the ICS cohort had also received ≥1 acute course of oral corticosteroids or had experienced exacerbations during the baseline period. Conversely, a higher proportion of patients in the non-ICS cohort were prescribed insulin before the index date. Characteristics were described using summary statistics and compared using t-tests, Mann-Whitney U tests, or Pearson’s χ^2^ tests. Patients were then matched in a 1:1 ratio based on pre-specified demographic characteristics, COPD severity and therapies, diabetes control, and potentially confounding comorbidities (Table 1, S2 Fig). A missing category was created for key variables to ensure no data was lost during matching.

**Table 2:** Key baseline demographic and clinical characteristics for unmatched and matched datasets comparing patients with COPD and type 2 diabetes on either ICS or non-ICS therapy. Additional baseline data can be found in S1 Table (S1 File).

Key patient characteristics		Unmatched		Matched	
		ICS (n = 1360)	non-ICS (n = 2642)*	ICS (n = 682)	non-ICS (n = 682)*
Age (years)	Mean (SD)	70.5 (9.3)	70.8 (9.1)	70.4 (7.8)	70.5 (7.7)
Age (categorised), n (%)	40–60	187 (13.8)	344 (13)	73 (10.7)	64 (9.4)
	61–80	980 (72.1)	1,914 (72.4)	549 (80.5)	553 (81.1)
	>80	193 (14.2)	384 (14.5)	60 (8.8)	65 (9.5)
Sex, n (%)	Male	911 (67)	1821 (68.9)	499 (73.2)	499 (73.2)
BMI, n (%)	Underweight	10 (0.7)	18 (0.7)	0 (0)	0 (0)
	Normal weight	215 (15.9)	394 (15)	67 (9.8)	67 (9.8)
	Overweight	428 (31.7)	813 (30.9)	222 (32.6)	222 (32.6)
	Obese	698 (51.7)	1409 (53.5)	393 (57.6)	393 (57.6)
Smoking status, n (%)^‡^	Non-smoker	141 (10.4)	265 (10.1)	31 (4.5)	31 (4.5)
	Current smoker	414 (30.6)	882 (33.7)	194 (28.4)	194 (28.4)
	Ex-smoker	800 (59.0)	1473 (56.2)	457 (67.0)	457 (67.0)
FEV_1_% predicted, n (%)^‡^	Missing	179 (13.2)	315 (11.9)	38 (5.6)	38 (5.6)
	<30 (very severe)	48 (3.5)	37 (1.4)	22 (3.2)	11 (1.6)
	30 –<50 (severe)	327 (24.0)	454 (17.2)	115 (16.9)	126 (18.5)
	50 –<80 (moderate)	645 (47.4)	1,514 (57.3)	416 (61.0)	426 (62.5)
	≥80 (mild)	161 (13.6)	322 (13.8)	91 (13.3)	81 (11.9)
mMRC score,[4] n (%)	Missing	55 (4)	112 (4.2)	4 (0.6)	4 (0.6)
	0–1	669 (49.2)	1429 (54.1)	383 (56.2)	383 (56.2)
	≥2	636 (46.8)	1101 (41.7)	295 (43.3)	295 (43.3)
Acute OCS courses, n (%)	0	1005 (73.9)	2232 (84.5)	545 (79.9)	585 (85.8)
	1	253 (18.6)	319 (12.1)	107 (15.7)	77 (11.3)
	≥2	102 (7.5)	91 (3.4)	30 (4.4)	20 (2.9)
Moderate/severe exacerbations^§^, n (%)	0	590 (43.4)	1402 (53.1)	346 (50.7)	387 (56.7)
	1	400 (29.4)	748 (28.3)	229 (33.6)	188 (27.6)
	≥2	370 (27.2)	492 (18.6)	107 (15.7)	107 (15.7)
HbA_1c_ (%, DCCT), n (%)	≤6.5%	458 (33.7)	809 (30.6)	214 (31.4)	197 (28.9)
	6.5 – ≤7.5%	531 (39.0)	1029 (38.9)	311 (45.6)	309 (45.3)
	>7.5%	371 (27.3)	804 (30.4)	157 (23.0)	176 (25.8)
Antidiabetic medication, n (%)	None	391 (28.7)	698 (26.4)	175 (25.7)	175 (25.7)
	Non-insulin	771 (56.7)	1492 (56.5)	424 (62.2)	424 (62.2)
	Insulin	198 (14.6)	452 (17.1)	83 (12.2)	83 (12.2)
GOLD group^¶^	Non-missing	1305 (96)	2530 (95.8)	678 (99.4)	678 (99.4)
	A, n (%)	362 (27.7)	969 (38.3)	257 (37.9)	256 (37.8)
	B, n (%)	292 (22.4)	684 (27.0)	186 (27.4)	187 (27.6)
	C, n (%)	307 (23.5)	460 (18.2)	126 (18.6)	127 (18.7)
	D, n (%)	344 (26.4)	417 (16.5)	109 (16.1)	108 (15.9)

*Includes prescriptions for SABA, SAMA, LABA, LAMA or their combinations. Patients may be included more than once with a different index date and prescription. Number of unique patients: 2,007.

^‡^Data recorded at or closest to the index date. Percentages within categories calculated from non-missing values only.

^§^Defined as the number of acute oral corticosteroid courses and antibiotic prescriptions for a lower respiratory tract infection.

^¶^Closest to the index date, see supplementary methods (S1 File) for allocation into categories.

BMI = body mass index; COPD = chronic obstructive pulmonary disorder; DCCT = Diabetes Control and Complications Trial units; FEV_1_ = forced expiratory volume in 1 second; GOLD = Global Initiative for Chronic Obstructive Lung Disease; HbA_1c_ = glycated haemoglobin; ICS = inhaled corticosteroids; LABA = long acting β_2_-agonist; LAMA = long acting muscarinic antagonist; mMRC = modified Medical Research Council; OCS = oral corticosteroids; SABA = short acting β_2_-agonist; SAMA = short acting muscarinic antagonist; SD = standard deviation.

The primary outcome (change in HbA_1c_ value) was compared using a generalised linear model, and results were reported as adjusted within- and between-group (ICS vs non-ICS) differences in change from baseline to outcome with 95% confidence interval. Binary outcomes were analysed using conditional logistic regression models and results were reported as odds ratios with 95% confidence intervals. Potential confounders (listed in S1 File), were checked for collinearity using Spearman’s correlation coefficients. Dose-dependent outcomes were investigated according to cumulative ICS dose exposure measured in fluticasone propionate equivalents (fluticasone propionate:extrafine beclomethasone dipropionate [BDP]:budenoside:non-extrafine BDP in a 1:1:2:2 ratio).[27–29] Time to progression to insulin in the outcome period was assessed using a Cox proportional hazards regression model. The proportional hazards assumption was checked before models were fitted. Analyses were carried out on the full data set of matched patients, as well as on a subgroup of patients with mild-to-moderate COPD, who were identified as belonging to GOLD groups A or B (more information on these groups can be found in S1 File). As this subgroup represents patients in which ICS prescribing may not be appropriate, the number needed to harm (NNH, 95% CI) was estimated, as the inverse of the risk difference. Finally, endpoints were compared in ICS cumulative dose subgroups (>125-250mg and >250mg, versus ≤125mg), to investigate a dose-dependent relationship. This analysis was carried out in the full study sample.

All potential confounding variables (listed in S1 File) were investigated in the final multivariate models, but were only included if they were significant in the model, or if their exclusion materially changed the main effect of interest. If a potential adjusting variable had strong co-linearity with another adjusting variable, the most significant was retained in the model. Analyses were performed using R statistical software (R Foundation for Statistical Computing, Austria), SPSS Statistics 22 (IBM SPSS Statistics, UK) and SAS 9.3 (SAS Institute, UK). Results were reported in accordance with STROBE guidelines for reporting cohort studies (S1 Checklist).

## Results

### Matched patient population

There were initially 1,360 eligible patients in ICS cohort and 2,642 eligible patients in the non-ICS cohort. After applying matching criteria, this was reduced to 682 unique patients in each cohort (S2 Fig and S1 File). 442 patients in each cohort had mild-to-moderate COPD (GOLD groups A and B), representing a similar proportion (65%) seen in previous data.[30] In the unmatched population, 28% of mild-to-moderate patients were prescribed ICS during the baseline period. The mean age of the full study sample was 70 years, and 73% were men; 95% of patients in each cohort were either current or ex-smokers, and 90% were either overweight or obese (Table 2). The spirometric severity of COPD was moderate in the majority of patients, with a %predicted FEV_1_ of 50–79 for 61% and 62.5% of patients in the ICS and non-ICS cohorts, respectively. The median (IQR) number of days between the baseline and last HbA_1c_ reading was 511 (386–596) and 526 (404–602) for ICS and non-ICS cohorts, respectively.

### Analysis of primary endpoint

**Fig 1:**
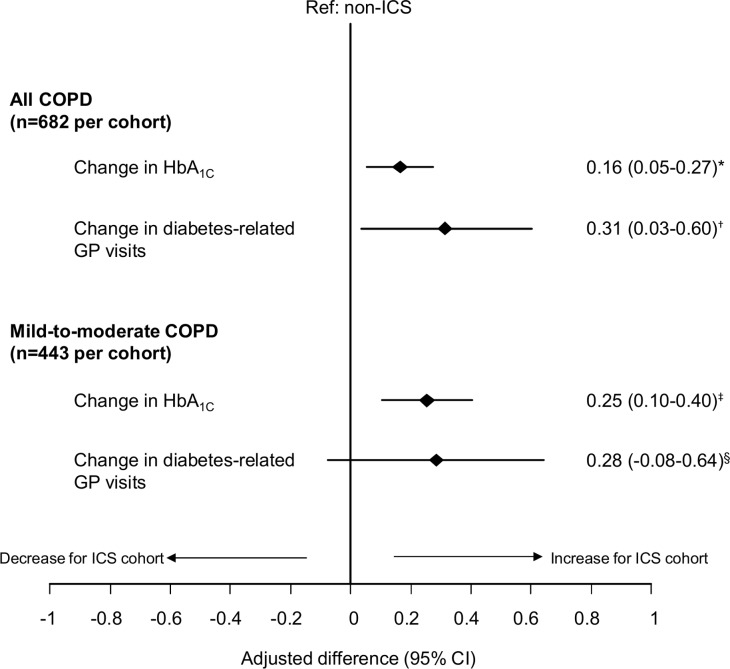
Comparison of changes in HbA_1C_ and diabetes-related GP visits between ICS and non-ICS cohorts. Mild-to-moderate COPD patients identified by GOLD groups A and B. *Adjusted for number of acute courses of OCS and diagnosis of pneumonia during baseline, time between baseline and outcome HbA_1c_ readings, and acute OCS use between index date and outcome HbA_1c_ readings. ^†^Adjusted for diagnosis of GORD in baseline, baseline HbA_1c_, and duration of diabetes. ^‡^Adjusted for number of acute OCS courses during baseline and time between baseline and outcome HbA_1c_ readings. ^§^Adjusted for baseline HbA_1c_ and duration of diabetes.CI = confidence interval; COPD = chronic obstructive pulmonary disease; HbA_1c_ = glycated haemoglobin; ICS = inhaled corticosteroids; GORD = gastro-oesophageal reflux disease; GOLD = Global Initiative for Chronic Obstructive Lung Disease; GP = general practice; OCS = oral corticosteroids.

Over the outcome period, an increase in HbA_1c_ value was observed in both the ICS and non-ICS cohorts (Table 3). However, patients prescribed ICS therapy experienced a significantly greater increase in HbA_1c_ from the baseline to the outcome period than those prescribed non-ICS therapies (Fig 1); relative to the non-ICS cohort, the adjusted difference in change in HbA_1c_ (95% CI) was 0.16% (0.05–0.27%) in the full study sample (all COPD patients). This difference was also significant, and larger, in the mild-to-moderate COPD patients (adjusted difference [95% CI], 0.25% [0.10–0.40%]).

**Table 3:** Study endpoints during the outcome year.

	ICS cohort	non-ICS cohort
Change in HbA_1c_ from baseline to outcome, median (IQR)	0.18 (-0.23, 0.60)	0.03 (-0.33, 0.50)
≥ 0.5 (DCCT %) increase in HbA_1c_ from baseline to outcome, n (%)	179 (29.9)	153 (25.5)
Addition of antidiabetic drug from baseline to outcome, n (%)	101 (16.9)	100 (16.7)
≥ 0.5 (DCCT %) increase in HbA_1c_ and/or addition of antidiabetic drug from baseline to outcome, n (%)	240 (40.1)	223 (37.2)
HbA_1c_ off QOF target (>7.5%), n (%)	209 (30.6)	194 (28.4)
Change in annual rate of diabetes-related GP visits, median (IQR)	0 (-1.01, 1.33)	-0.01 (-1.34, 1)
Increase in diabetes-related hospitalisation rate, n (%)	41 (6.0)	31 (4.5)
Increase in prescription rate for glucose strips, n (%)	114 (16.7)	75 (11.0)
Progression to insulin, n (%)	28 (4.7)	12 (2.0)

DCCT = Diabetes Control and Complications Trial units; GP = general practice; HbA_1c_ = glycated haemoglobin; ICS = inhaled corticosteroids; IQR = interquartile range; OCS = oral corticosteroids; QOF = Quality and Outcomes Framework.

### Analysis of secondary endpoints

**Fig 2:**
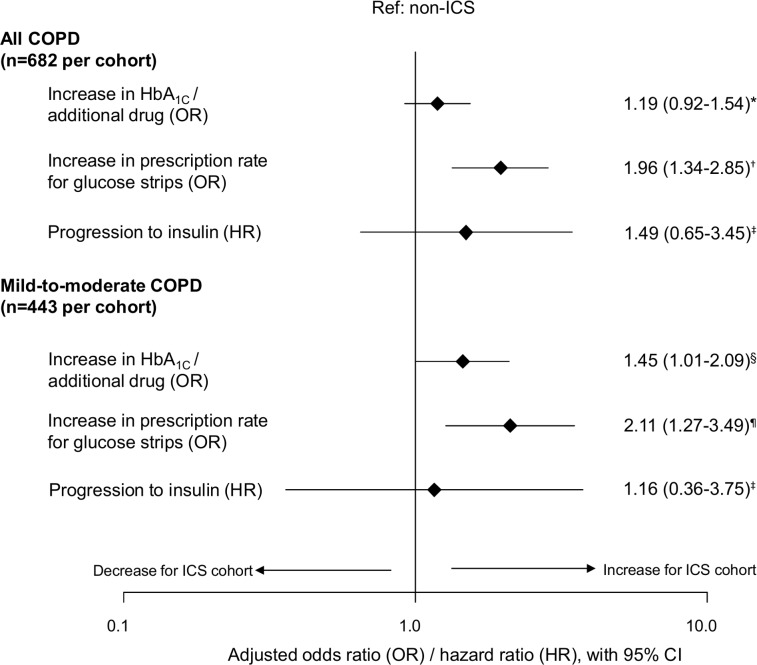
Comparison of other diabetes-related outcomes between ICS and non-ICS cohorts. Mild-to-moderate COPD patients identified by GOLD groups A and B. *Adjusted for diagnosis of GORD in baseline, duration of diabetes, time between baseline and outcome HbA_1c_ readings, and acute OCS use between index date and outcome HbA_1c_ readings. ^†^ Adjusted for number of allergy prescriptions and number of primary care consultations in baseline. ^‡^ Adjusted for baseline antidiabetic medication. ^§^Adjusted for number of COPD consultations in baseline, time between baseline and outcome HbA_1c_ readings, and acute OCS use between index date and outcome HbA_1c_ readings. ^¶^Adjusted for number of GP consultations in baseline. CI = confidence interval; COPD = chronic obstructive pulmonary disease; HbA_1c_ = glycated haemoglobin; ICS = inhaled corticosteroids; GORD = gastro-oesophageal reflux disease; GOLD = Global Initiative for Chronic Obstructive Lung Disease; GP = general practice; OCS = oral corticosteroids.

The change in diabetes-related GP visits was significantly higher in the ICS cohort versus the non-ICS cohort, in the full study sample (adjusted difference [95% CI], 0.31 [0.03–0.60]), but not in the mild-to-moderate COPD patients (Fig 1). ICS-treated patients were significantly more likely to increase the prescription rate for glucose strips from baseline to outcome period, compared to non-ICS patients (Fig 2). This was found in all COPD patients and in the mild-to-moderate subgroup. However, the adjusted odds of having an increase in HbA_1c_ value and/or an additional antidiabetic drug was only significant in the mild-to-moderate COPD patients (odds ratio [95% CI], 1.45 [1.10–2.09], Fig 2).

A higher but non-significant proportion of patients in the ICS cohort who were prescribed two or more types of non-insulin antidiabetic medication during the baseline year progressed to insulin during the outcome period compared with those in the non-ICS cohort (unadjusted proportions; S3 Fig). After confirming a valid proportional hazards assumption (S4 Fig), the time to progression to insulin showed no significant differences between patients prescribed ICS vs non-ICS therapies (Fig 2). This could be due to the low number of patients progressing to insulin (n = 28 and n = 12 for ICS and non-ICS cohorts, respectively. There was also no difference in progression to insulin in the mild-to-moderate COPD subgroup. No significant differences were observed in other measures of T2DM progression, including the number of patients not reaching the QOF target for HbA_1c_ (ie HbA_1c_ >7.5%), or an increase in diabetes-related hospitalisation rates (Table 3).

### The effects of ICS dose

**Fig 3:**
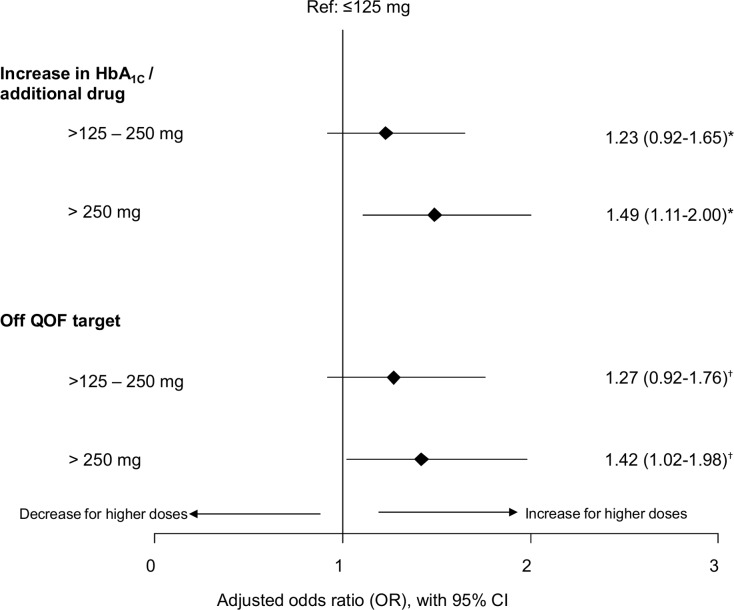
Adjusted odds ratios of the effect of cumulative ICS dose exposure and measured in fluticasone equivalents from the first prescription at the index date to the last HbA_1c_ value in the outcome period. *Adjusted for baseline HbA_1c_ and acute oral corticosteroid use between index date and outcome HbA_1c_. ^†^Adjusted for duration of diabetes (combined first diagnosis and first prescription), baseline HbA_1c_ and acute oral corticosteroid use between index date and outcome HbA_1c_. HbA_1c_ = glycated haemoglobin; ICS = inhaled corticosteroids.

The adjusted odds of having a ≥0.5 (DCCT %) increase in HbA_1c_ value and/or additional antidiabetic medication were significantly greater for patients with a cumulative ICS dose exposure of >250 mg (fluticasone equivalents) in the outcome period compared with those with lower doses (≤125 mg). Patients with higher ICS dose exposure also experienced significantly greater odds of being above QOF target for HbA_1c_ (HbA_1c_ >7.5%) in the outcome period compared with those on lower doses (Fig 3). Results on time to insulin progression are not presented in this case as the proportional hazards assumption was not valid. For patients prescribed >250 mg ICS, the number needed to harm, NNH (95% CI), was estimated to be 11 (6.2–39) compared with patients prescribed ≤125 mg ICS. Thus, for every 11 patients prescribed >250 mg, 1 patient would have an increased risk of an increase in HbA_1c_ and/or receiving additional antidiabetic medication who would not have had this if prescribed ≤125 mg.

## Discussion

### Principal findings

In this historical matched cohort study, patients with COPD and comorbid T2DM who were prescribed ICS experienced a significant negative impact on diabetes control in terms of increase in HbA_1c_ levels, diabetes-related general practice visits, and prescriptions of glucose strips as compared with matched patients not prescribed ICS. Some effects were stronger in mild-to-moderate COPD patients; an important finding, given that these patients should not receive ICS according to treatment guidelines. Patients prescribed high doses of ICS were also at greater risk of diabetes progression than those prescribed lower doses. These results were demonstrated after adjustment for important clinical characteristics.

### Clinical relevance

Despite finding significant differences in change in HbA_1c_ levels between ICS and non-ICS cohorts, these differences were small. Adjusted differences were 0.16% (ICS vs non-ICS) in the full data set, and 0.25% in mild-moderate COPD patients. However, it should be noted that, in the case of the mild-moderate patients, this difference was demonstrated in spite of the ICS cohort being more likely to receive additional drugs to control their diabetes during the outcome period. It should also be noted that the observed outcome period varied between 12 and 18 months, due to the nature of the real-life data source. The average follow-up time was 1.4 years: if the study had measured HbA_1c_ change over one year, the observed change would be approximately 0.11% (0.16/1.4). It is possible that such small changes accumulating over a longer time period could be clinically important, impacting on quality of life or the risk of long-term diabetes complications, as discussed by McQueen et al[31]. This issue calls for further research to investigate the long-term effects.

### Strengths and limitations

This study drew on two large high-quality data sources, the CPRD and OPCRD, allowing us to obtain a sizeable sample of a specific patient population with comorbid COPD and T2DM. These data bases are well described and have previously been used in respiratory research.[19, 32] The primary data outcome, HbA_1c_ value, is a reliable and clinically important marker of glucose control in diabetes mellitus.[33, 34] We used an observational cohort design, which reflects the real-life prescribing activity in our study population. A randomised controlled trial (RCT), although more rigorous in reducing bias in the comparison of treatment groups, would not be practicable in this scenario. It would be unethical to randomise a severe COPD patient to no ICS therapy, when ICS is the recommended treatment in national guidelines. For the mild-moderate COPD patients, ICS therapy is not recommended. Yet because this recommendation is frequently ignored, we were able to use observational data to make a clinically important comparison between ICS and no ICS in this group of patients. Even so, as there is no randomisation procedure in an observational study, systematic differences may arise between two cohorts. In this study, we aimed to minimise these differences through matching of important demographic and clinical characteristics, and through adjustment by other potential confounders. In particular, we ensured that variables that were likely to influence the level of HbA1c, such as baseline HbA1c, antidiabetic medication and acute oral corticosteroid use, were investigated as confounding factors. Despite these methods, there remains the possibility of residual bias due to unmeasured confounders. It is unknown exactly why a GP may choose to prescribe ICS to one patient while another GP prescribes no ICS to a similar patient. The impact of such confounding, however, may be less in the mild-moderate group of our study, where treatment decisions are more subjective. It was in this group that we observed the strongest negative effect of ICS. Our use of exact matching in the study design resulted in a large reduction in sample size from 1,360 and 2,642 eligible patients to 685 matched patients in each cohort. We accepted this reduction in order to maximise the balance of characteristics between the two cohorts. However, the exclusion of patients may have made the final study sample less representative of the patient population and therefore may affect the generalisability of our results. The exclusion of patients due to a lack of HbA_1c_ measurement may also contribute to a decrease in generalisability. Further, to increase the number of patients that could be matched we increased particular category sizes of matching variables. For example, we used an age category of 70–75, so it should be noted that our analysis may have included comparisons between 70 year olds and 75 year olds. Such patients may differ in many ways, but we have matched by important factors to maximise comparability.

Our primary endpoint captured the change in HbA_1c_ over a period of 12–18 months. What is not clear is whether the bulk of the change occurred at a particular point, or was gradual over time. This is an interesting research question and should be considered for future study. In addition, larger studies are recommended to detect a wider range of effect sizes.

### Strengths and limitations in relation to other studies

In agreement with previous studies,[17, 18, 35, 36] we found that prescribing of ICS is not restricted to patients with severe airflow limitation and/or those with two or more exacerbations per year as recommended by GOLD guidelines. Of the eligible patients with mild to moderate disease severity (GOLD groups A and B), 28% were prescribed ICS during the baseline period. These patients were at increased risk for reduced glycaemic control during the outcome period. However, previously published data regarding the effects of ICS on glucose metabolism are mixed.[37] Randomised controlled trials of ICS treatment in patients with COPD did not identify increased rates of adverse diabetes-related events. This could be because most of these trials relied on spontaneous adverse events rather than focusing on diabetes control. These studies may therefore not have been powered to detect diabetes-related parameters, and they may not have studied long enough outcome periods.[8, 26, 38–40] Conversely, real-life studies have indicated significant detrimental effects of ICS on diabetes control.[6, 12] Our results broadly agree with those of Suissa et al.,[6] who reported that ICS use was associated with an increased risk of diabetes progression. We also found that patients with higher ICS dose exposure are at significantly greater risk of diabetes progression, as assessed by both clinically relevant increases in HbA_1c_ and/or prescription of additional antidiabetic medication, higher odds of HbA_1c_ values not meeting the QOF target, (HbA_1c_ >7.5%), and increased glucose strip prescriptions.

### Meaning of the study

As evidenced by numerous studies, ICS use can have substantial adverse effects that need to be weighed against the expected benefits for specific patients or patient groups.[9, 10, 41–44] In the case of patients with COPD and T2DM, our results indicate that ICS therapy is associated with a negative impact on diabetes control, and this effect increases with higher ICS doses. Although ICS may be beneficial for reducing exacerbations in patients with severe COPD, [38, 45–47] the current guidelines for COPD management do not indicate ICS treatment for patients with less severe disease (GOLD groups A and B). Even so, a high proportion of these patients are currently prescribed ICS, thereby exposing them to additional risks without benefits, something that might be particularly relevant in a population where 90% of patients are either overweight or obese. Our findings highlight the importance of multifactorial risk assessment for patients with COPD, considering their risk factors for diabetes and other common comorbidities. Further research with longer outcome periods is required to determine the effect of ICS use and cumulative dose on time to progression to insulin. Further research may also consider a prospective cohort study in place of a retrospective study. Here, investigators could arrange to capture even more measurements that may potentially confound the results, and not have to rely only on the measurements that exist in historical data.

### Conclusions

In conclusion, our results suggest that prescribing of ICS for patients with COPD and comorbid T2DM is associated with a negative impact on diabetes control, and patients with higher ICS dose exposure may be at a greater risk of diabetes progression than those with lower exposure. Increased awareness of COPD management guidelines is required to avoid putting this patient group at unnecessary risk, and to assist patients and healthcare providers in making informed decisions.

## Supporting Information

S1 Checklist STROBE 2007 (v4) Statement—Checklist of items that should be included in reports of cohort studies (DOCX)

S1 Fig Patient flow chart. * Received a prescription for ICS (ICS cohort) or received a prescription for SABA, SAMA, LABA or LAMA (non-ICS cohort). ^†^Same date of birth, sex, index date and prescription at index date. COPD = chronic obstructive pulmonary disease; HbA1c = glycated haemoglobin; ICS = inhaled corticosteroids; LABA = long-acting β_2_-agonist, LAMA = long-acting muscarinic antagonist; SABA = short-acting β_2_-agonist; SAMA = short-acting muscarinic antagonist.
(DOCX)

S2 Fig Matching flow chart. * Received a prescription for ICS (ICS cohort) or received a prescription for SABA, SAMA, LABA or LAMA (non-ICS cohort). BMI = body mass index; FEV_1_ = forced expiratory volume in 1 second; HbA1c = glycated haemoglobin; ICS = inhaled corticosteroids; LABA = long-acting β_2_-agonist, LAMA = long-acting muscarinic antagonist; mMRC = modified Medical Research Council score; SABA = short-acting β_2_-agonist; SAMA = short-acting muscarinic antagonist.
(DOCX)

S3 Fig Proportion of patients in the ICS and non-ICS therapy cohorts who progressed to insulin during the outcome period, relative to their prescription of non-insulin antidiabetic drugs during the baseline period. **Percentages are relative to the total number of patients in each baseline category.** ICS = inhaled corticosteroids.
(DOCX)

S4 Fig Survival functions showing time to progression to insulin in the outcome period. ICS = inhaled corticosteroids.
(DOCX)

S1 File Online supplementary material. Supplementary methods.
(DOCX)

S1 Protocol Study protocol. (PDF)

S1 Table Additional baseline demographic and clinical characteristics for the unmatched dataset comparing patients with COPD and type 2 diabetes on either ICS or non-ICS therapy. *Within 1 year prior to the index date. ^†^Average daily dose within 1 year prior to the index date, calculated as ([count of inhalers × doses in pack]/365) × μg strength. ^‡^Recorded any time prior to the index date. ^§^Estimated as the difference between the date of first diagnosis and the index date. ^¶^Estimated as the difference between the date of first prescription of antidiabetic medication (after a full year of practice data) and the index date. ^#^Last value recorded prior to the index date. ^**^Total per drug class. COPD = chronic obstructive pulmonary disease; DCCT = Diabetes Control and Complaints Unit; FEV_1_ = forced expiratory volume in 1 second; HbA1c = glycated haemoglobin; ICS = inhaled corticosteroids; IFCC = International Federation of Clinical Chemistry; IQR = interquartile range; LABA = long acting β_2_-agonist; LAMA = long acting muscarinic antagonist; QOF = Quality and Outcomes Framework; SABA = short acting β_2_-agonist; SAMA = short acting muscarinic antagonist.
(DOCX)
