# Supplementary material for: Metabolic Effects Associated with ICS in Patients with COPD and Comorbid Type 2 Diabetes: A Historical Matched Cohort Study
Source: PLoS One. 2016 Sep 22;11(9):e0162903. doi: 10.1371/journal.pone.0162903 (PMC5033451; doi:10.1371/journal.pone.0162903)
Supplement: S1 Checklist — (DOCX) [file pone.0162903.s001.docx]

**STROBE 2007 (v4) Statement—Checklist of items that should be included in reports of *cohort studies***

| **Section/Topic** | Item # | Recommendation | Reported on page # |
| --- | --- | --- | --- |
| **Title and abstract** | 1 | (*a*) Indicate the study’s design with a commonly used term in the title or the abstract | Title page – page 1 |
|  |  | (*b*) Provide in the abstract an informative and balanced summary of what was done and what was found | Methods and findings section of abstract – Page 2–3 |
| Introduction | | |  |
| Background/rationale | 2 | Explain the scientific background and rationale for the investigation being reported | Introduction – Pages 4-5 |
| Objectives | 3 | State specific objectives, including any prespecified hypotheses | Introduction – Page 5 |
| Methods | | |  |
| Study design | 4 | Present key elements of study design early in the paper | Methods – Page 5–6 |
| Setting | 5 | Describe the setting, locations, and relevant dates, including periods of recruitment, exposure, follow-up, and data collection | Methods section of abstract – Page 2,  Methods – Pages 6–7 |
| Participants | 6 | (*a*) Give the eligibility criteria, and the sources and methods of selection of participants. Describe methods of follow-up | Methods – Page 6–7, Supplementary Figure 1 |
|  |  | (*b*) For matched studies, give matching criteria and number of exposed and unexposed | Table 1, Supplementary Figure 2 |
| Variables | 7 | Clearly define all outcomes, exposures, predictors, potential confounders, and effect modifiers. Give diagnostic criteria, if applicable | Study endpoints – Page 7, Table 1, Supplementary methods – Pages 2–4 of supplementary material |
| Data sources/ measurement | 8* | For each variable of interest, give sources of data and details of methods of assessment (measurement). Describe comparability of assessment methods if there is more than one group | Data Source – Page 6 |
| Bias | 9 | Describe any efforts to address potential sources of bias | Supplementary materials – Page 3–4, Figure S2 |
| Study size | 10 | Explain how the study size was arrived at | Figure S1 |
| Quantitative variables | 11 | Explain how quantitative variables were handled in the analyses. If applicable, describe which groupings were chosen and why | Methods – Page 5-7 |
| Statistical methods | 12 | (*a*) Describe all statistical methods, including those used to control for confounding | Methods – Pages 10–14 |
|  |  | (*b*) Describe any methods used to examine subgroups and interactions | Methods – Page 14 |
|  |  | (*c*) Explain how missing data were addressed | Methods – Page 10 |
|  |  | (*d*) If applicable, explain how loss to follow-up was addressed | N/A |
|  |  | (*e*) Describe any sensitivity analyses | N/A |
| Results | | |  |
| Participants | 13* | (a) Report numbers of individuals at each stage of study—eg numbers potentially eligible, examined for eligibility, confirmed eligible, included in the study, completing follow-up, and analysed | Figures S1, S2, Results – Pages 14–15 |
|  |  | (b) Give reasons for non-participation at each stage | Figures S1, S2 |
|  |  | (c) Consider use of a flow diagram | Figures S1, S2 |
| Descriptive data | 14* | (a) Give characteristics of study participants (eg demographic, clinical, social) and information on exposures and potential confounders | Tables 2, S1 |
|  |  | (b) Indicate number of participants with missing data for each variable of interest | Table 2 |
|  |  | (c) Summarise follow-up time (eg, average and total amount) | N/A (defined on methods page 7) |
| Outcome data | 15* | Report numbers of outcome events or summary measures over time | Results – Page 15–19 Table 3, Figure 1A |
| Main results | 16 | (*a*) Give unadjusted estimates and, if applicable, confounder-adjusted estimates and their precision (eg, 95% confidence interval). Make clear which confounders were adjusted for and why they were included | Results – Page 18 Table 3, Figure 1A |
|  |  | (*b*) Report category boundaries when continuous variables were categorized | Table 3 |
|  |  | (*c*) If relevant, consider translating estimates of relative risk into absolute risk for a meaningful time period | N/A |
| Other analyses | 17 | Report other analyses done—eg analyses of subgroups and interactions, and sensitivity analyses | Results – Page 18–19, Figure 1A, 1B |
| Discussion |  |  |  |
| Key results | 18 | Summarise key results with reference to study objectives | Discussion – Page 21 |
| **Limitations** |  |  |  |
| Interpretation | 20 | Give a cautious overall interpretation of results considering objectives, limitations, multiplicity of analyses, results from similar studies, and other relevant evidence | Pages 21–23 |
| Generalisability | 21 | Discuss the generalisability (external validity) of the study results | Pages 21, 22 |
| Other information |  |  |  |
| Funding | 22 | Give the source of funding and the role of the funders for the present study and, if applicable, for the original study on which the present article is based | Page 24 |

*Give information separately for cases and controls in case-control studies and, if applicable, for exposed and unexposed groups in cohort and cross-sectional studies.

**Note:** An Explanation and Elaboration article discusses each checklist item and gives methodological background and published examples of transparent reporting. The STROBE checklist is best used in conjunction with this article (freely available on the Web sites of PLoS Medicine at http://www.plosmedicine.org/, Annals of Internal Medicine at http://www.annals.org/, and Epidemiology at http://www.epidem.com/). Information on the STROBE Initiative is available at www.strobe-statement.org.
