# Supplementary material for: Metabolic Effects Associated with ICS in Patients with COPD and Comorbid Type 2 Diabetes: A Historical Matched Cohort Study
Source: PLoS One. 2016 Sep 22;11(9):e0162903. doi: 10.1371/journal.pone.0162903 (PMC5033451; doi:10.1371/journal.pone.0162903)
Supplement: S1 Fig — * Received a prescription for ICS (ICS cohort) or received a prescription for SABA, SAMA, LABA or LAMA (non-ICS cohort). †Same date of birth, sex, index date and prescription at index date. COPD = chronic obstructive pulmonary disease; HbA1c = glycated haemoglobin; ICS = inhaled corticosteroids; LABA = long-acting β2-agonist, LAMA = long-acting muscarinic antagonist; SABA = short-acting β2-agonist; SAMA = short-acting muscarinic antagonist. (DOCX) [file pone.0162903.s002.docx]

**S1 Figure:** Patient flow chart

All patients with type 2 diabetes initiating therapy* within the study period (2008–2012)

(n = 12 528 / 33 523)

HbA_1c_ data available

(n = 2245 / 6888)

Type 2 diabetes diagnosis before ICS prescription

(n = 9611 / 25,865)

Received a type 2 diabetes diagnosis code after initiating on ICS (n = 2917 / 7658)

Valid baseline period

(n = 8154 / 22 338)

COPD diagnosis

(n = 2670 / 8123)

Joined practice less than 12 months before first ICS prescription (n = 1457 / 3527)

No COPD diagnosis code (n = 5484 /
14 215)

Age ≥40 years

(n = 2665 / 8110)

Aged <40 years at index date (n = 5 / 13)

No or invalid HbA_1c_ data (n = 420 / 1222)

**Final eligible patients**

**(n = 1360 / 2642)**

Valid ICS adherence

(n = 1445 / 2835)

No maintenance oral corticosteroids

(n = 1394 / 2740)

Adherence to ICS in the outcome period <50%

(n = 800 / 4053)

Prescriptions for maintenance oral corticosteroids in the baseline period

(n = 51 / 95)

**ICS cohort / non-ICS cohort**

**Exclusion criteria**

Duplicates removed (n = 34 / 98)^†^

* Received a prescription for ICS (ICS cohort) or received a prescription for SABA, SAMA, LABA or LAMA (non-ICS cohort).
^†^Same date of birth, sex, index date and prescription at index date.
COPD = chronic obstructive pulmonary disease; HbA1c = glycated haemoglobin; ICS = inhaled corticosteroids; LABA = long-acting β_2_-agonist, LAMA = long-acting muscarinic antagonist; SABA = short-acting β_2_-agonist; SAMA = short-acting muscarinic antagonist.
