# Supplementary material for: Metabolic Effects Associated with ICS in Patients with COPD and Comorbid Type 2 Diabetes: A Historical Matched Cohort Study
Source: PLoS One. 2016 Sep 22;11(9):e0162903. doi: 10.1371/journal.pone.0162903 (PMC5033451; doi:10.1371/journal.pone.0162903)
Supplement: S2 Fig — * Received a prescription for ICS (ICS cohort) or received a prescription for SABA, SAMA, LABA or LAMA (non-ICS cohort). BMI = body mass index; FEV1 = forced expiratory volume in 1 second; HbA1c = glycated haemoglobin; ICS = inhaled corticosteroids; LABA = long-acting β2-agonist, LAMA = long-acting muscarinic antagonist; mMRC = modified Medical Research Council score; SABA = short-acting β2-agonist; SAMA = short-acting muscarinic antagonist. (DOCX) [file pone.0162903.s003.docx]

**S2 Fig:** Matching flow chart

* Received a prescription for ICS (ICS cohort) or received a prescription for SABA, SAMA, LABA or LAMA (non-ICS cohort)
BMI = body mass index; FEV_1_ = forced expiratory volume in 1 second; HbA1c = glycated haemoglobin; ICS = inhaled corticosteroids; LABA = long-acting β_2_-agonist, LAMA = long-acting muscarinic antagonist; mMRC = modified Medical Research Council score; SABA = short-acting β_2_-agonist; SAMA = short-acting muscarinic antagonist.
