# Supplementary material for: Metabolic Effects Associated with ICS in Patients with COPD and Comorbid Type 2 Diabetes: A Historical Matched Cohort Study
Source: PLoS One. 2016 Sep 22;11(9):e0162903. doi: 10.1371/journal.pone.0162903 (PMC5033451; doi:10.1371/journal.pone.0162903)
Supplement: S3 Fig — Percentages are relative to the total number of patients in each baseline category. ICS = inhaled corticosteroids. (DOCX) [file pone.0162903.s004.docx]

**S3 Fig:** Proportion of patients in the ICS and non-ICS therapy cohorts who progressed to insulin during the outcome period, relative to their prescription of non-insulin antidiabetic drugs during the baseline period. Percentages are relative to the total number of patients in each baseline category.

ICS = inhaled corticosteroids.
