# Supplementary material for: Metabolic Effects Associated with ICS in Patients with COPD and Comorbid Type 2 Diabetes: A Historical Matched Cohort Study
Source: PLoS One. 2016 Sep 22;11(9):e0162903. doi: 10.1371/journal.pone.0162903 (PMC5033451; doi:10.1371/journal.pone.0162903)
Supplement: S4 Fig — ICS = inhaled corticosteroids. (DOCX) [file pone.0162903.s005.docx]

**S4 Fig:** Survival functions showing time to progression to insulin in the outcome period.


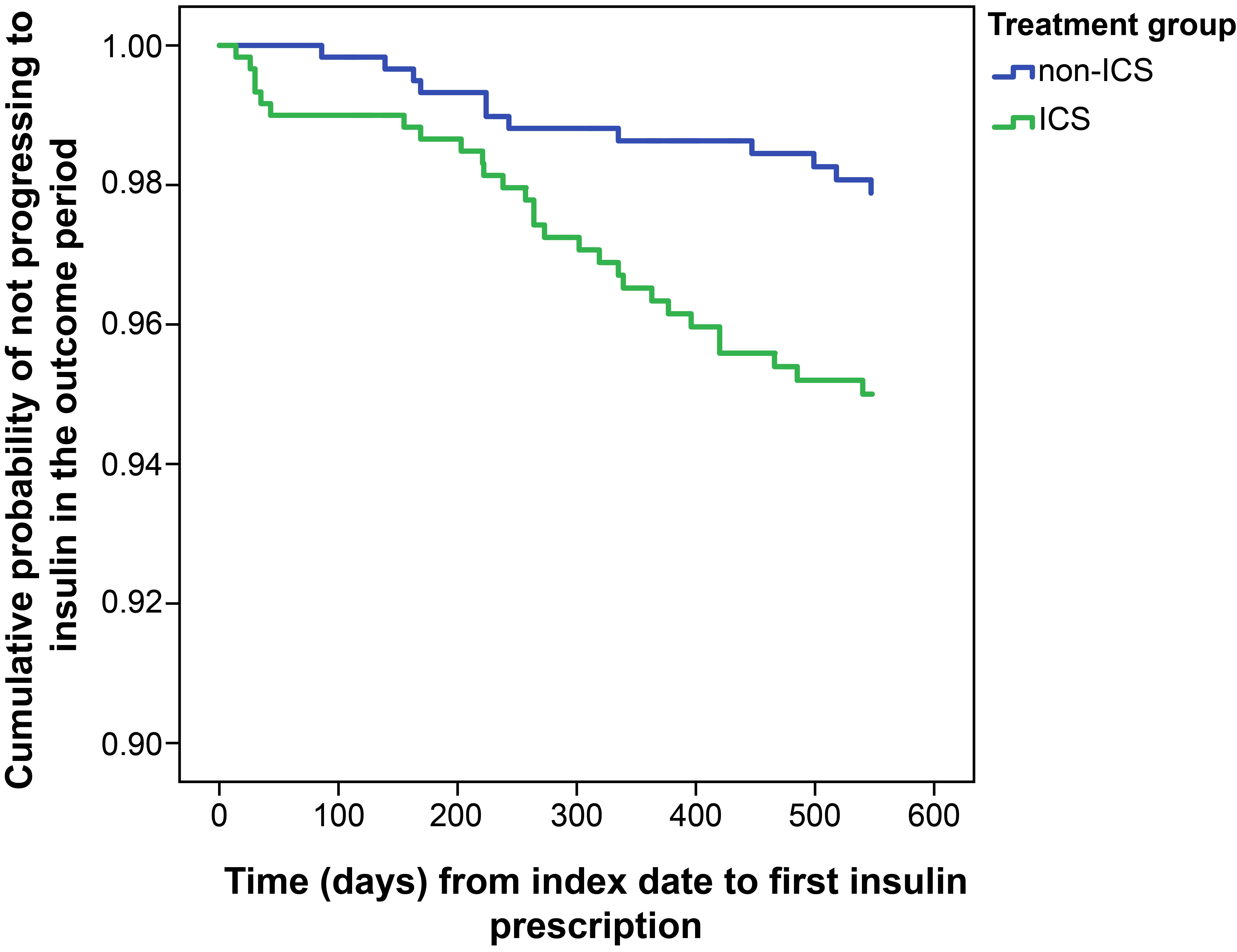


ICS = inhaled corticosteroids.
