# Supplementary material for: Metabolic Effects Associated with ICS in Patients with COPD and Comorbid Type 2 Diabetes: A Historical Matched Cohort Study
Source: PLoS One. 2016 Sep 22;11(9):e0162903. doi: 10.1371/journal.pone.0162903 (PMC5033451; doi:10.1371/journal.pone.0162903)
Supplement: S1 File — Supplementary methods. (DOCX) [file pone.0162903.s006.docx]

## Online supplementary material

**Metabolic effects associated with ICS in patients with COPD and comorbid type 2 diabetes: a historical matched cohort study**

David B Price, Richard Russell, Rafael Mares, Anne Burden, Derek Skinner, Helga Mikkelsen, Richard Brice, Niels H Chavannes, Janwillem WH Kocks, Jeffrey W Stephens, John Haughney

## Supplementary methods

### Codes use to select patient population

*COPD included*

| **Read Code** | **Definition** |
| --- | --- |
| H3... | Chronic obstructive pulmonary disease |
| H3... | Chronic obstructive airways disease |
| H31.. | Chronic bronchitis |
| H310. | Simple chronic bronchitis |
| H3100 | Chronic catarrhal bronchitis |
| H3101 | Smokers' cough |
| H310z | Simple chronic bronchitis NOS |
| H311. | Mucopurulent chr.bronchitis |
| H3110 | Purulent chronic bronchitis |
| H3111 | Fetid chronic bronchitis |
| H311z | Mucopurulent chr.bronchit.NOS |
| H312. | Obstructive chronic bronchitis |
| H3120 | Chronic asthmatic bronchitis |
| H3120 | Chronic wheezy bronchitis |
| H3121 | Emphysematous bronchitis |
| H3122 | Acute exacerbation of COAD |
| H3123 | Bronchiolitis obliterans |
| H312z | Obstructive chr.bronchitis NOS |
| H313. | Mixd simp+mucopur chron bronch |
| H31y. | Other chronic bronchitis |
| H31y0 | Chronic tracheitis |
| H31y1 | Chronic tracheobronchitis |
| H31yz | Other chronic bronchitis NOS |
| H31z. | Chronic bronchitis NOS |
| H32.. | Emphysema |
| H320. | Chronic bullous emphysema |
| H3200 | Segmental bullous emphysema |
| H3201 | Zonal bullous emphysema |
| H3202 | Giant bullous emphysema |
| H3203 | Bullous emphysema + collapse |
| H3203 | Tension pneumatocoele |
| H320z | Chronic bullous emphysema NOS |
| H321. | Panlobular emphysema |
| H322. | Centrilobular emphysema |
| H32y. | Other emphysema |
| H32y0 | Acute vesicular emphysema |
| H32y1 | Atrophic (senile) emphysema |
| H32y1 | Acute interstitial emphysema |
| H32y2 | MacLeod's unilateral emphysema |
| H32yz | Other emphysema NOS |
| H32yz | Sawyer - Jones syndrome |
| H32z. | Emphysema NOS |
| H36.. | Mild chron obstr pulm disease |
| H37.. | Mod chron obstr pulm disease |
| H38.. | Sev chron obstr pulm disease |
| H39.. | Very severe COPD |
| H3y.. | Chronic obstr.airway dis.OS |
| H3y.. | OS chron obstruct pulmon dis |
| H3y0. | Chr obs pulm dis+ac l resp inf |
| H3y1. | Chr obs pulm dis+ac exac,unspc |
| H3z.. | Chronic obstr.airway dis.NOS |
| H3z.. | Chr obstruc pulmonary dis NOS |
| H4640 | Chronic chemical emphysema |
| H4641 | Chemical obliter.bronchiolitis |
| H582. | Compensatory emphysema |
| Hyu30 | [X]Other emphysema |
| Hyu31 | [X]O spcf chron obs pulmon dis |
| X101j | Occupational chr bronchitis |
| X101k | Byssinosis grade 3 |
| X101m | Drug-induced bronchiolit oblit |
| X101n | Pulmonary emphysema |
| X101o | Pulm emphysema, alpha-1 PI def |
| X101p | Toxic emphysema |
| X101q | Congenital lobar emphysema |
| X101r | Scar emphysema |
| X102z | Bronchiolitis oblit with UIP |
| XE0ZN | Chronic: [bronchitis NOS] or++ |
| XaIND | End stag chron obst airway dis |
| XaIQg | Interstit pulmonary emphysema |

*Type II diabetes included*

| **Read Code** | **Definition** |
| --- | --- |
| 66Ao.00 | Diabetes type 2 review |
| 66A3.00 | Diabetic on diet only |
| 66A4.00 | Diabetic on oral treatment |
| 66AV.00 | Diabetic on insulin+oral treat |
| C100112 | Non-insulin depend.diabet.mell |
| C109.00 | Non-insulin depd diabetes mell |
| C109.11 | NIDDM - Non-insu dep diab mel |
| C109.12 | Type 2 diabetes mellitus |
| C109.13 | Type II diabetes mellitus |
| C109000 | Non-ins-dp diab mel+renal comp |
| C109011 | Type II diab mell renal compl |
| C109012 | Type 2 diab mell renal compl |
| C109100 | Non-ins-dp diab mel+ophth comp |
| C109111 | Type II diab mell ophthal comp |
| C109112 | Type 2 diab mell ophthal comp |
| C109200 | Non-ins-dp diab mel+neuro comp |
| C109211 | Type II diab mell neurol comp |
| C109212 | Type 2 diab mell neurol comp |
| C109300 | Non-ins-dp diab mel+multi comp |
| C109311 | Type II diab mell multip comp |
| C109312 | Type 2 diab mell multip comp |
| C109400 | Non-insul depen diab mel+ulcer |
| C109411 | Type II diab mell with ulcer |
| C109412 | Type 2 diab mell with ulcer |
| C109500 | Non-insulin dep diab mell+gang |
| C109511 | Type II diab mell + gangrene |
| C109512 | Type 2 diab mell + gangrene |
| C109600 | Non-insul dep diab mel+retinop |
| C109611 | Type II diab mell retinopathy |
| C109612 | Type 2 diab mell retinopathy |
| C109700 | Non-insul dep diab-poor contr |
| C109711 | Type II diab mell poor control |
| C109712 | Type 2 diab mell poor control |
| C109900 | Non-insul-dep diab mel no comp |
| C109911 | Type II diab mell no complic |
| C109912 | Type 2 diab mell no complic |
| C109A00 | NIDDM with mononeuropathy |
| C109A11 | Type II diab mell mononeurop |
| C109A12 | Type 2 diab mell mononeurop |
| C109B00 | NIDDM with polyneuropathy |
| C109B11 | Type II diab mell polyneurop |
| C109B12 | Type 2 diab mell polyneurop |
| C109C00 | NIDDM with nephropathy |
| C109C11 | Type II diab mell nephropathy |
| C109C12 | Type 2 diab mell nephropathy |
| C109D00 | NIDDM with hypoglycaemic coma |
| C109D11 | Type II diab mell hypogly coma |
| C109D12 | Type 2 diab mell hypogly coma |
| C109E00 | NIDDM with diabetic cataract |
| C109E11 | Type II diab mell diab catarct |
| C109E12 | Type 2 diab mell diab catarct |
| C109F11 | Type II diab mell perip angiop |
| C109F12 | Type 2 diab mell perip angiop |
| C109G00 | NIDDM with arthropathy |
| C109G11 | Type II diab mell arthropathy |
| C109G12 | Type 2 diab mell arthropathy |
| C109H11 | Type II diab mell neurop arthr |
| C109H12 | Type 2 diab mell neurop arthr |
| C109J00 | Insul treated Type 2 diab mell |
| C109J11 | Ins treat non-ins dep diab mel |
| C109J12 | Insul treat Type II diab mell |
| C109K00 | Hyperos non-ket stat typ 2 d m |
| C10F.00 | Type 2 diabetes mellitus |
| C10F.11 | Type II diabetes mellitus |
| C10F000 | Type 2 diab mell + renal compl |
| C10F011 | Type II diab mell renal compl |
| C10F100 | Type 2 diab mell+ophthal comp |
| C10F111 | Type II diab mell ophthal comp |
| C10F200 | Type 2 diab mell + neurol comp |
| C10F211 | Type II diab mell neurol comp |
| C10F300 | Type 2 diab mell + multip comp |
| C10F311 | Type II diab mell multip comp |
| C10F400 | Type 2 diab mell with ulcer |
| C10F411 | Type II diab mell with ulcer |
| C10F500 | Type 2 diab mell + gangrene |
| C10F511 | Type II diab mell + gangrene |
| C10F600 | Type 2 diab mell + retinopathy |
| C10F611 | Type II diab mell retinopathy |
| C10F700 | Type 2 diab mell+poor control |
| C10F711 | Type II diab mell poor control |
| C10F900 | Type 2 diab mell without comp |
| C10F911 | Type II diab mell without comp |
| C10FA00 | Type 2 diab mell mononeurop |
| C10FA11 | Type II diab mell mononeurop |
| C10FB00 | Type 2 diab mell + polyneurop |
| C10FB11 | Type II diab mell polyneurop |
| C10FC00 | Type 2 diab mell + nephropathy |
| C10FC11 | Type II diab mell nephropathy |
| C10FD00 | Type 2 diab mell+hypogly coma |
| C10FD11 | Type II diab mell hypogly coma |
| C10FE00 | Type 2 diab mell+diab catarct |
| C10FE11 | Type II diab mell diab catarct |
| C10FF00 | Type 2 diab mell+perip angiop |
| C10FF11 | Type II diab mell perip angiop |
| C10FG00 | Type 2 diab mell + arthropathy |
| C10FG11 | Type II diab mell+arthropathy |
| C10FH00 | Type 2 diab mell neurop+arthr |
| C10FH11 | Type II diab mell neurop+arthr |
| C10FJ00 | Insul treated Type 2 diab mell |
| C10FJ11 | Insul treat Type II diab mell |
| C10FK00 | Hyperos non-ket stat typ 2 d m |
| C10FL00 | Type 2 d m + persist proteinur |
| C10FL11 | Type II d m + persist protein |
| C10FM00 | Type 2 d m + persist microalb |
| C10FN00 | Type 2 d m with ketoacidosis |
| C10FP00 | Type 2 d m+ketoacidotic coma |
| C10FQ00 | Type 2 d m + exudat maculopath |
| C10FR00 | Type 2 dm with gastroparesis |
| C10FM00 | Type 2 d m + persist microalb |
| C10FM11 | Type II d m + persist microalb |
| ZC2CA00 | Dietary advice for type II diabetes |

*Type II diabetes included*

| **Read Code** | **Definition** |
| --- | --- |
| C10FS00 | Matern inherited diabetes mell |

### Measures of COPD severity

Chronic obstructive pulmonary disease (COPD) severity was assessed during the baseline period using the forced expiratory volume in 1 second (FEV_1_) and the modified Medical Research Council (mMRC) score closest to the index date. FEV_1_ was measured via spirometry and expressed as a percentage of the predicted normal value. The mMRC score assesses breathlessness, graded from 0 (lowest score) to 4 (severe breathlessness).^1^ Patients were then divided into groups A–D according to the symptom and exacerbation risk management model of the Global Initiative for Chronic Obstructive Pulmonary Disease (GOLD), 2014.^1^ Allocation into GOLD groups closest to the index date was carried out according to the following criteria:

**Group A** (low risk, fewer symptoms)

- mMRC score ≤1 AND
- FEV_1_ ≥50% AND/OR
- ≤1 exacerbation per year with no hospitalisations for exacerbations

**Group B** (low risk, more symptoms)

- mMRC score ≥2 AND
- FEV_1_ ≥50% AND/OR
- ≤1 exacerbation per year with no hospitalisations for exacerbations

**Group C** (high risk, fewer symptoms)

- mMRC score ≤1 AND
- FEV_1_ <50% AND/OR
- ≥2 exacerbations per year or ≥1 hospitalisation for exacerbation

**Group D** (high risk, more symptoms)

- mMRC score ≥2 AND
- FEV_1_ <50% AND/OR
- ≥2 exacerbations per year or ≥1 hospitalisation for exacerbation

### Potential confounders examined

*Demographic characteristics* **(**at or closest to the index date)

- Age
- Sex
- Body mass index (BMI)
- Smoking status

*Measures of COPD severity and COPD-related therapies*

- Lung function measurements (FEV_1_)
- GOLD group classification† closest to the index date
- Moderate and severe COPD exacerbations within the baseline period‡
- COPD-related therapies prescribed within the year prior to index date§
- All acute oral corticosteroid courses prescribed in baseline period and prior to baseline HbA_1c_

*Measures of diabetic control* (during baseline period)

- Duration of medication-treated diabetes
- HbA_1c_ value prior to index date
- Antidiabetic medication and glucose strip prescriptions
- Diabetes-related hospitalisations

*Comorbidities*

- Asthma
- Cardiovascular disease
- Ischaemic heart disease
- Hypertension
- Charlson Comorbidity Index score for the year prior to the index date

## References

1. Global Initiative for Chronic Obstructive Lung Disease. Global strategy for the diagnosis, management and prevention of chronic obstructive pulmonary disease 2015. <http://www.goldcopd.org/guidelines-global-strategy-for-diagnosis-management.html>

2. British Medical Association and NHS England. 2016/16 General Medical Services (GMS) contract Quality and Outcomes Framework (QOF). Guidance for GMS contract 2015/16. March 2015. [http://bma.org.uk/support-at-work/contracts/gp-contracts-and-funding/independent-contractors/qof-guidance](%20http:/bma.org.uk/support-at-work/contracts/gp-contracts-and-funding/independent-contractors/qof-guidance).
