# Supplementary material for: Metabolic Effects Associated with ICS in Patients with COPD and Comorbid Type 2 Diabetes: A Historical Matched Cohort Study
Source: PLoS One. 2016 Sep 22;11(9):e0162903. doi: 10.1371/journal.pone.0162903 (PMC5033451; doi:10.1371/journal.pone.0162903)
Supplement: S1 Protocol — (PDF) [file pone.0162903.s007.pdf]

Metabolic effects associated with ICS (inhaled corticosteroid) use in COPD (chronic obstructive pulmonary disease) patients with type II diabetes

A retrospective observational evaluation of adverse metabolic consequences associated with (appropriate and inappropriate) ICS use in COPD patients with type II diabetes

Boehringer Ingelheim Team:

Anna Scowcroft
Outcomes Research Team Manager. Market Access Pricing & Outcomes Research

Dr Lisa White
Medical and Scientific Affairs Manager. Medical and Scientific Affairs

Research in Real Life (RiRL) Team:

David Price
Professor of Primary Care Respiratory Medicine, University of Aberdeen, and Director of RiRL

Rafael Mares
Project Researcher

Annie Burden
Project Statistician

Derek Skinner
Project Data Manager

Emily Davis
Research Coordinator

Catherine Hutton
Chief Executive Officer

Lay Summary

Current international guidelines for the management of chronic obstructive pulmonary disease (COPD; GOLD 2014) recommend inhaled corticosteroids (ICS) are reserved for COPD patients with severe/very severe disease and/or frequent exacerbations. However, research shows widespread use of ICS in patients with mild and moderate disease, meaning more patients are exposed to risks of side effects than would be expected under current guidelines. High doses of ICS as those typically prescribed to COPD patients have been linked to increased risks of diabetes onset and progression, yet no study has investigated this association in a cohort of COPD patients in the UK. The proposed study will investigate whether ICS treatment in COPD patients with comorbid type II diabetes has a negative impact on diabetic control, and determine whether COPD patients treated with ICS outside of guidelines are at unnecessary risk of diabetes progression.

Contents

BACKGROUND 3
Aim and Objectives 4
Primary objective 4
Secondary objectives 4
Study cohorts 4
METHODS 4
Study type and design 4
Study outcomes 5
Primary outcome: 5
Secondary outcomes: 5
Study population 5
Inclusion criteria: 5
Data source 6
Sample size justification 7
Data analysis 8
Primary outcome: 8
Secondary outcomes: 9
Internal and external validity 9
Strengths and possible limitations 10
Plans for disseminating and communicating study results 10
Patient/user group involvement 10
TIMELINE 11
REFERENCES 11

BACKGROUND

Current international guidelines for the management of chronic obstructive pulmonary disease (COPD; GOLD 2014)[1] recommend long-acting inhaled bronchodilators, including β_2-agonists (LABA) and anti-muscarinic agents (LAMA) as maintenance therapies. These agents can be prescribed as a monotherapy or in combination with inhaled corticosteroids (ICS) for the symptomatic management of COPD and the prevention of exacerbations.

GOLD management guidelines[1] recommend ICS are reserved for COPD patients with severe/very severe disease and/or frequent exacerbations (groups C and D; Figure 1). However, despite significant efforts to promote and disseminate the guidelines, they have not been widely implemented by primary care physicians or respiratory physicians in secondary care, resulting in a significant dissociation between guideline recommendations and clinicians' practices[2,3]. Indeed, research shows widespread use of ICS in patients in GOLD groups A and B[4], meaning more patients are exposed to risks of side effects than would be expected under current guidelines.

Figure 1. GOLD patient groups

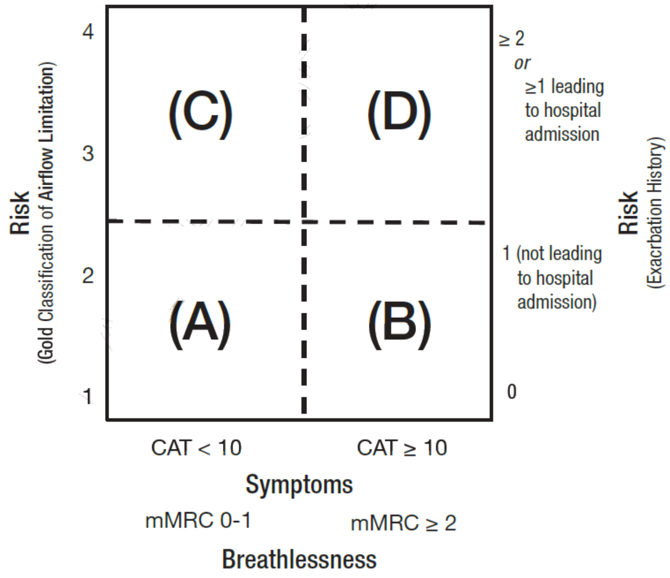

GOLD Classification of Airflow Limitation: 1 (mild), 2 (moderate), 3 (severe), 4 (very severe). CAT: COPD Assessment Test. mMRC: modified British Medical Research Council questionnaire. Figure adapted from GOLD 2014 guidelines[1].

COPD patients are particularly susceptible to the potential side effects of ICS due to:

- older age (~ ≥ 40 years)[1,5]
- high rates of obesity/inactivity[6,7]
- often prescribed higher doses of ICS than asthma patients, with pivotal trials involving doses of 1,000 µg of fluticasone per day for 2-3 years[8,9]

The potential link between ICS use and diabetes progression in COPD patients is currently of great interest, but to date, there has been no study investigating this association in a cohort of COPD patients in the UK. Recent evidence from a study of over 350,000 patients in Canada suggests that high-dose ICS effects may include increased risks of diabetes onset and progression[7]. In this study, the risks were found to be more pronounced at the high ICS doses prescribed in COPD patients.

The proposed study will investigate whether ICS treatment in COPD patients with comorbid type II diabetes has a negative impact on diabetic control, and determine whether COPD patients treated with

ICS outside of guidelines are at unnecessary risk of diabetes progression. It will form the basis from which COPD patients with comorbid type II diabetes are informed of the risks associated with initiating ICS therapy and are presented with an alternative management therapy. The intended audience for this study is prescribers. We plan to publish the results of this study initially at a conference, then as a manuscript in a peer-reviewed journal.

Aim and Objectives

We propose to conduct an evaluation of the potential adverse metabolic consequences associated with ICS use in COPD patients with comorbid type II diabetes.

Primary objective

To assess whether ICS use (within and outside of GOLD guidelines) is associated with an increase in glycated haemoglobin (HbA_{1c}) value (%) in COPD patients with type II diabetes

Secondary objectives

- Assess whether ICS use is associated with an increase in HbA_{1c} and change in anti-diabetic medication
- Assess whether ICS use is associated with an increase in the number of patients off HbA_{1c} target (< 7.5% as per UK QOF indicators[†])
- Assess whether ICS use is associated with increases in GP visits, hospital visits and glucose strip use
- Assess whether ICS use is associated with the progression of ongoing diabetes treatment to insulin
- Assess whether ICS dose is associated with diabetes progression
- Assess whether patients who should not be receiving ICS treatment according to GOLD guidelines (groups A and B) are at risk of diabetes progression

Study cohorts

Patients with COPD and comorbid type II diabetes initiating ICS treatment will be compared to a control cohort of similar patients not treated with ICS and initiating LABA or LAMA treatment only.

METHODS

Study type and design

This will be a retrospective cohort study with a one year outcome period after an index prescription date (Figure 2). The index prescription date refers to either the first prescription of ICS (ICS-initiating cohort) or the first prescription of LABA or LAMA (no ICS therapy cohort). The cohort study design was chosen because it has been identified that it may be difficult to find enough patients in the non-ICS group, due to the fact that ICS is such a commonly prescribed treatment in this patient population. Using a cohort design will ensure that the study will contain enough non-ICS patients to meet the requirements of the power calculation.

[†] Quality and Outcomes Framework; NICE indicator published in 2010.

Figure 2. Study design

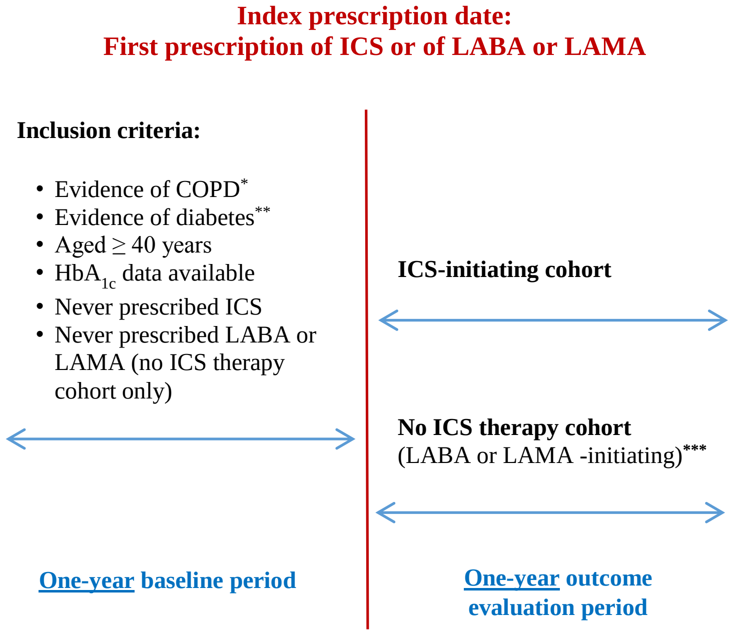

* COPD diagnostic read code ever recorded plus spirometry measurement within 5 years of the index date confirming the diagnosis (i.e. individuals with a FEV_1/FVC ratio < 0.7).

** Diagnostic read code for type II diabetes. Patients with any record of type I diabetes will be excluded.

*** Patients in the two treatment arms (i.e. ICS-initiating and no ICS therapy cohorts) may be matched on important baseline, clinical characteristics, such as: age ± 5 years, sex, BMI, short-acting β_2 agonist use, smoking status, COPD disease severity and diabetes duration.

Study outcomes

Primary outcome:

- Change in HbA_{1c} value (%) relative to baseline

Secondary outcomes:

- Change in HbA_{1c} with a change in anti-diabetic medication
- Change in number of patients on/off HbA_{1c} target (< 7.5% as per UK QOF indicators) with no change in anti-diabetic medication
- No change in HbA_{1c} with an increase in GP visits, hospital visits and glucose strip use
- Progression of ongoing diabetes treatment to insulin

Primary and secondary study outcomes apply to the analyses of the effects of both ICS use and ICS dose, and to the analyses on a subset of patients who should not be receiving ICS treatment according to GOLD guidelines, as per the objectives listed above (see details in data analysis methods below).

Study population

Inclusion criteria:

- A diagnosis of COPD prior to index date (COPD diagnostic read code ever recorded plus spirometry measurement within five years of the index date confirming the diagnosis, i.e. individuals with a FEV_1/FVC ratio < 0.7)

- A diagnosis of type II diabetes prior to index date (diagnostic read code for type II diabetes; patients with any record of type I diabetes will be excluded)
- Aged ≥ 40 years at index date (based on GOLD review of prevalence of COPD)
- HbA_{1c} data available within the year prior to index date, and between 20 days and one year post index date
- At least one full year of data prior to and after the index date
- For the ICS-initiating cohort, a first ever prescription for ICS between 2008 and 2012, with a Medication Possession Ratio (MPR)[10] ≥ 50% for any dose of ICS within the outcome period.
- For the no ICS therapy cohort, a first ever prescription for LABA or LAMA between 2008 and 2012, and no ICS therapy prior to or within the outcome period (i.e. before or within the year after the index date). Patients will only be considered if they have at least two prescriptions of LABA or LAMA within the outcome period (may be relaxed to one prescription, depending on patient numbers)[‡].

Patients in the ICS-initiating cohort will be matched to patients in the no ICS therapy cohort (i.e. control cohort). Patients may be matched on:

- Age ± 5 years at index date
- Sex
- Baseline BMI
- Short-acting β_2 agonist use (average daily dose during baseline)[§]
- Smoking status
- COPD disease severity (GOLD criteria)
- Baseline HbA_{1c}
- Duration of diabetes at index date

Data source

This study is to be conducted using the Optimum Patient Care Research Database (OPCRD) and data from the Clinical Practice Research Datalink (CPRD) in order to ensure sufficient patient numbers. OPCRD and CPRD data are widely used in clinical, epidemiological and pharmaceutical research[**], and the specific use of UK primary care databases to study COPD has been validated in previous studies[11].

The OPCRD is an anonymised longitudinal database with a focus on respiratory diseases that includes data from over 400 UK General Practices. Use of the OPCRD data has been approved by the Trent Multi Centre Research Ethics Committee for clinical research use. All applications to use the database are reviewed by the Independent Anonymous Data Ethics Protocols and Transparency (ADEPT) Committee[††].

The CPRD contains the primary care medical records from more than 660 UK GP practices, including diagnoses, prescriptions, referrals and test results. It covers approximately 6% of UK patients, and the geographical distribution is representative of the UK population. Use of CPRD data requires

‡ If an insufficient number of patients initiating on LABA or LAMA is found for the control cohort, patients receiving a first ever prescription of SABA or SAMA may be included (previous RiRL research suggests no major differences in COPD severity between patients prescribed short/long -acting medication and ICS therapy). All other matching criteria will apply.
§ See previous footnote ‡.
** OPCRD, http://www.optimumpatientcare.org/Html_Docs/OPCRD.html; CPRD, http://www.cprd.com/Bibliography/
†† The protocol was submitted to ADEPT on 19/06/2014 and is currently under review.

submission of the study protocol to the Independent Scientific Advisory Committee (ISAC)[‡‡] at the Medicines and Healthcare Products Regulatory Agency (MHRA).

Combining the OPCRD and CPRD data: OPCRD and CPRD data will initially be analysed separately. Once summarised datasets have been created, a 'fuzzy' matching process will be performed in which patients are checked for whether year of birth, sex, index dates, and dates of first diagnosis for the main comorbidities are the same across datasets. If this is found to be the case, duplicated patients will be dropped from the final dataset. At no stage in this process will patient data be deanonymised. Moreover, the RiRL team carrying out the analysis, have experience in combining these datasets[12] and there is no risk of patients being deanonymised during the course of the study.

Sample size justification

To detect a difference in the mean changes in HbA_{1c} of 0.25% (based on a study by Faul et al. 2009[§§]), assuming that the common standard deviation is 1.1 (based on RiRL unpublished data), the following sample sizes have been calculated (using a two group t-test with a 0.05 two-sided significance level):

For 90% power (based on matched ratios of 1:1, 2:1 and 3:1):

	**Matching ratio**		
	**1:1**	**1:2**	**1:3**
**Non ICS**	408	306	272
**ICS**	408	612	816

For 80% power (based on matched ratios of 1:1, 2:1 and 3:1):

	**Matching ratio**		
	**1:1**	**1:2**	**1:3**
**Non-ICS**	305	229	204
**ICS**	305	458	612

For 90% power and a matched ratio of 3:1, a minimum of 816 individuals treated with ICS (meeting the inclusion criteria) are required. The OPCRD contains 705 individuals and the CPRD is estimated to hold data for 1370 individuals meeting the inclusion criteria (based on the number of individuals per practice obtained from the OPCRD). Using both databases will ensure we have a large enough sample size to detect differences in mean changes in HbA_{1c} of 0.25%.

‡‡ The protocol was submitted to ISAC on 18/06/2014 and is currently under review.
§§ Faul et al. (2009) carried out a small pilot study evaluating the impact of initiation of fluticasone propionate versus montelukast on diabetic control in patients with asthma or COPD[13]. The authors reported mean changes in HbA_{1c} from baseline of 0.11% and -0.14% in the ICS and non-ICS groups. This resulted in a small but significant difference of 0.25% over six weeks of follow up.

Data analysis

Exploratory data analysis will be performed first. This enables baseline and outcome variables to be checked, for example, for validity of data, missing data and outliers.

Unmatched baseline data analysis will then be carried out in line with the statistical epidemiological analysis plan. Following this analysis, the matching criteria will be discussed further with the sponsor team including the epidemiologist on the project and the statistical analysis plan will be revised if necessary (e.g. if not enough patients are found for the control cohort). This step takes place before the outcomes have been analysed, and is therefore not influenced by the results.

To ensure the comparison of like patients, individual patients in the two treatment arms (i.e. ICS-initiating cohort and no ICS therapy cohort) may be matched on important clinical characteristics, such as:

- Age ± 5 years at index date
- Sex
- Baseline BMI
- Short-acting β_2 agonist use (average daily dose during baseline)
- Smoking status
- COPD disease severity (GOLD criteria)
- Baseline HbA_{1c}
- Duration of diabetes at index date
- Insulin at baseline

These baseline characteristics will be summarized and compared between the two groups. Other covariates which may considered in the analysis include:

- Duration of COPD at index date
- Cardiovascular disease, ischemic heart disease and hypertension
- Other comorbidities expressed using the Charlson Comorbidity Index (CCI)
- Anti-diabetic therapies (other than insulin)
- Diabetes-related hospitalizations

For all analyses detailed below, differences between the study cohorts will be considered statistically significant when $p < 0.05$, and considered to be showing a trend when $p < 0.10$. Changes in $HbA_{1c} \geq 0.5\%$ will be considered clinically significant. Results will be presented with their 95% confidence intervals.

Primary outcome:

- Change in HbA_{1c} relative to baseline

For the primary outcome, the baseline HbA_{1c} (within the year prior to index date) and first HbA_{1c} reading that is > 20 days post index date will be used. The maximum period for HbA_{1c} readings that will be evaluated will be one year post initiation of treatment (ICS or control).

Mean one-year change in HbA_{1c} value (%) will be compared across the two study cohorts using a two-group two-sided t-test. If the interval between the baseline HbA_{1c} reading and the index date is not roughly the same length as the interval between the index date and the post treatment HbA_{1c} reading, then we will adjust in the analysis for the time between the two HbA_{1c} measurements.

Secondary outcomes:

- Change in HbA_{1c} with a change in anti-diabetic medication

For this outcome, the baseline HbA_{1c} (within the year prior to index date) and first HbA_{1c} reading that is > 20 days post index date will be used. The maximum period for HbA_{1c} readings that will be evaluated will be one year post initiation of treatment (ICS or control). The differences between ICS treated and control cohorts in the number of patients that suffered an increase in HbA_{1c} and changed anti-diabetic medication post index date will be analysed.

- Change in number of patients on/off HbA_{1c} target (< 7.5% as per UK QOF indicators) with no change in anti-diabetic medication

For this outcome, the baseline HbA_{1c} (within the year prior to index date) and HbA_{1c} reading closest to one year post index date will be used. The differences between ICS treated and control cohorts in change in number of patients off HbA_{1c} target with no change in anti-diabetic medication will be analysed.

- No change in HbA_{1c} with an increase in GP visits, hospital visits and glucose strip use

Frequency of GP visits, hospital visits and glucose strip use in the year pre and the year post index date will be used to determine whether they are affected by ICS use.

- Progression of ongoing diabetes treatment to insulin

Using a subset of patients who have not been prescribed insulin before the index date, the effect of ICS treatment on time to progression to insulin will be assessed using survival analysis. Time to insulin will be defined as time from first prescription for ICS (ICS treatment cohort) or for LABA or LAMA (control cohort) to a first prescription for insulin. Patients will be censored at the end of the one year outcome period.

Effects of ICS dose on diabetes progression: the effects of ICS dose on both primary and secondary outcomes will also be investigated, focusing on patients in the ICS-initiating cohort. ICS dose refers to the average daily dose of ICS prescriptions (converted to fluticasone equivalents) in the one year outcome period. Patients will be grouped by average daily dose as follows: high: ≥ 1,000 µg/day; moderate: 500-999 µg/day; low: < 500 µg/day (based on Suissa et al. 2010[7]). Similar analyses to those described above for each outcome (primary and secondary) will be carried out, but in this case, ICS dose rather than use will be considered.

Risk of diabetes progression in patients who should not be receiving ICS treatment according to GOLD guidelines (groups A and B): additional sensitivity analyses exploring the effects of ICS use on both primary and secondary outcomes will be carried out on a subset of patients who fit the following criteria: do not have a comorbid asthma diagnosis, experience less than 2 exacerbations (not leading to hospital admission) in the year prior to the index date, and have an $FEV_1 \geq 50$. Analyses similar to those described above for each outcome (primary and secondary) will be carried out.

Internal and external validity

Internal validity will be strived for, first by matching the ICS exposed and unexposed cohorts on a number of variables which are believed to be potential confounders. In addition, other potential confounders will be adjusted for within each analysis.

To validate the exposure variable of ICS use, we will ensure that patients were indeed exposed to ICS therapy by requiring that patients have an MPR of at least 50% for any dose within the one year

outcome period. Patients who received only one prescription cannot be assumed to have been exposed to ICS and will not be included in the study.

To validate the diagnosis of COPD required for a patient to be included in the study, diagnostic read codes will be combined with spirometry results.

To validate the diagnosis of type II diabetes we will compare individuals with a diabetic read code and those with anti-diabetic drugs prescribed to explore the robustness of the coding.

External validity will be achieved by using large primary care databases (i.e. the OPCRD and CPRD), which have been shown to be generalizable to the UK population.

Strengths and possible limitations

As with all observational studies, there are some potential limitations which we will attempt to address.

First, the data comes from an existing database, which means that the time points at which measurements are taken are not controlled, but are simply in line with the usual course of treatment of the patient. Therefore HbA_{1c} measurements may not occur at the same time points across patients. To address this, we will set limits to when HbA_{1c} measurements were recorded relative to the index prescription date in order for a patient to be included in the study.

Second, there are a number of variables which could potentially confound the relationship between ICS use and progression of diabetes. We will adjust for a number of these by matching the two cohorts, in order to ensure that the groups are balanced with respect to the most important confounders. Other potential confounders, such as comorbidity of cardiovascular disease, will be adjusted for in each analysis.

Third, there is a potential issue with misclassification bias, in that if a patient had only one prescription for ICS they might be classified as exposed to ICS when in fact they never dispensed or used the medication. To ensure that this does not bias our results, only patients with an MPR of at least 50% for any dose of ICS within the outcome year will be included in the ICS treated cohort. Patients who received only one prescription cannot be assumed to have been exposed and will not be included in the study.

Finally, as the data sources selected for this study are primary care databases it is likely that there will be missing data, where certain variables were not collected in the course of routine care. To address this, we will exclude patients who are missing important variables such as HbA_{1c} measurements. This should not be a major issue, because baseline characteristics, such as age, sex and all comorbidities, are reliably recorded by GPs in primary care databases.

Plans for disseminating and communicating study results

We intend to publish our findings in a peer-reviewed journal and also to present them at relevant scientific conferences.

Patient/user group involvement

We do not believe this research would benefit from patient group involvement at this stage, although we will actively collaborate with such groups in the dissemination strategy. It is possible that future research may well benefit from such involvement.

TIMELINE

Milestone	Expected date
Data extraction	July 2014
Data analysis	July – August 2014
Preliminary results	August 2014
Final data report	October 2014
Manuscript submission	December 2014

REFERENCES

1. Global Strategy for the Diagnosis, Management and Prevention of COPD, Global Initiative for Chronic Obstructive Lung Disease (GOLD) 2014.

2. Roche N, et al. Guidelines versus clinical practice in the treatment of chronic obstructive pulmonary disease. *Eur Respir J* 2001; 18(6):903-908.

3. Seaman J, et al. Health care utilization history, GOLD guidelines, and respiratory medication prescriptions in patients with COPD. *Int J Chron Obstruct Pulmon Dis* 2010; 5:89-97.

4. Corrado A & Rossi A. How far is real life from COPD therapy guidelines? An Italian observational study. *Respir Med* 2012; 106(7): 989-997.

5. Decramer M, et al. Clinical trial design considerations in assessing long-term functional impacts of tiotropium in COPD: the UPLIFT trial. *COPD* 2004; 1(2): 303-312.

6. Franssen FME, et al. Obesity and the lung: 5 · Obesity and COPD. *Thorax* 2008; 63(12):1110-1117.

7. Suissa S, et al. Inhaled corticosteroids and the risks of diabetes onset and progression. *Am J Med* 2010; 123(11):1001-1006.

8. Calverley PM, et al. Salmeterol and fluticasone propionate and survival in chronic obstructive pulmonary disease. *N Engl J Med* 2007; 356(8):775-789.

9. Wedzicha JA, et al. The prevention of chronic obstructive pulmonary disease exacerbations by salmeterol/fluticasone propionate or tiotropium bromide. *Am J Respir Crit Care Med* 2008; 177:19-26.

10. Peterson AM, et al. A checklist for medication compliance and persistence studies using retrospective databases. *Value Health* 2007; 10(1):3-12

11. Soriano JB, et al. Validation of general practitioner-diagnosed COPD in the UK General Practice Research Database. *Eur J of Epidemiol* 2002; 17:1075-1080.

12. Price D, et al. Switching patients from other inhaled corticosteroid devices to the Easyhaler®: historical, matched-cohort study of real-life asthma patients. *J Asthma Allergy* 2014; 7:31-51.

13. Faul JL, et al. 2009. The effect of an inhaled corticosteroid on glucose control in type 2 diabetes. *Clin Med Res* 7:14–20.
