# Supplementary material for: Metabolic Effects Associated with ICS in Patients with COPD and Comorbid Type 2 Diabetes: A Historical Matched Cohort Study
Source: PLoS One. 2016 Sep 22;11(9):e0162903. doi: 10.1371/journal.pone.0162903 (PMC5033451; doi:10.1371/journal.pone.0162903)
Supplement: S1 Table — *Within 1 year prior to the index date. †Average daily dose within 1 year prior to the index date, calculated as ([count of inhalers × doses in pack]/365) × μg strength. ‡Recorded any time prior to the index date. §Estimated as the difference between the date of first diagnosis and the index date. ¶Estimated as the difference between the date of first prescription of antidiabetic medication (after a full year of practice data) and the index date. #Last value recorded prior to the index date. **Total per drug class. COPD = chronic obstructive pulmonary disease; DCCT = Diabetes Control and Complaints Unit; FEV1 = forced expiratory volume in 1 second; HbA1c = glycated haemoglobin; ICS = inhaled corticosteroids; IFCC = International Federation of Clinical Chemistry; IQR = interquartile range; LABA = long acting β2-agonist; LAMA = long acting muscarinic antagonist; QOF = Quality and Outcomes Framework; SABA = short acting β2-agonist; SAMA = short acting muscarinic antagonist. (DOCX) [file pone.0162903.s008.docx]

## Supplementary table

**S1 Table**

Additional baseline demographic and clinical characteristics for the unmatched dataset comparing patients with COPD and type 2 diabetes on either ICS or non-ICS therapy.

|  |  | **ICS**  **(n = 1360)** | **non-ICS**  **(n = 2642)** | **p-value** |
| --- | --- | --- | --- | --- |
| FEV_1_ % predicted  (closest to index date) | Non-missing | 1181 (86.8) | 2327 (88.1) | - |
|  | Median (IQR) | 58.2 (46, 71.8) | 62 (51.5, 73) | <0.001 |
| COPD exacerbations | Median (IQR) | 1 (0, 2) | 0 (0, 1) | <0.001 |
| COPD exacerbations, n (%) | 0 | 590 (43.4) | 1402 (53.1) | <0.001 |
|  | 1 | 400 (29.4) | 748 (28.3) |  |
|  | 2 | 207 (15.2) | 322 (12.2) |  |
|  | ≥3 | 163 (12) | 170 (6.4) |  |
| GOLD group, n (%) | Non-missing | 1305 (96) | 2530 (95.8) | - |
|  | A | 362 (27.7) | 969 (38.3) | <0.001 |
|  | B | 292 (22.4) | 684 (27) |  |
|  | C | 307 (23.5) | 460 (18.2) |  |
|  | D | 344 (26.4) | 417 (16.5) |  |
| Antibiotic prescriptions (baseline period) | 0 | 684 (50.3) | 1509 (57.1) | <0.001 |
|  | 1 | 348 (25.6) | 657 (24.9) |  |
|  | 2 | 165 (12.1) | 295 (11.2) |  |
|  | ≥3 | 163 (12) | 181 (6.9) |  |
| COPD therapy^*^, n (%) | None | 463 (34) | 1739 (65.8) | <0.001 |
|  | SABA (+/-SAMA) | 520 (38.2) | 636 (24.1) |  |
|  | LABA (+/-SABA, +/-SAMA) | 59 (4.3) | 63 (2.4) |  |
|  | LAMA (+/-SABA, +/-SAMA) | 251 (18.5) | 126 (4.8) |  |
|  | LABA/LAMA (+/-SABA, +/-SAMA) | 37 (2.7) | 5 (0.2) |  |
|  | Other | 30 (2.2) | 73 (2.8) |  |
| SABA inhaler usage  (µg per day^†^), n (%) | 0 | 590 (43.4) | 1914 (72.4) | <0.001 |
|  | ≤200 | 443 (32.6) | 429 (16.2) |  |
|  | 201–400 | 173 (12.7) | 185 (7) |  |
|  | 401–800 | 126 (9.3) | 99 (3.7) |  |
|  | >800 | 28 (2.1) | 15 (0.6) |  |
| Asthma diagnosis^‡^, n (%) | | 87 (6.4) | 57 (2.2) | <0.001 |
| Other chronic respiratory diseases^‡^, n (%) | | 22 (1.6) | 29 (1.1) | 0.215 |
| Gastro-oesophageal reflux disease^*^, n (%) | | 10 (0.7) | 42 (1.6) | 0.034 |
| Cardiovascular disease^‡^, n (%) | | 818 (60.1) | 1617 (61.2) | 0.539 |
| Ischaemic heart disease^‡^, n (%) | | 614 (45.1) | 1189 (45) | 0.958 |
| Heart failure^‡^, n (%) | | 167 (12.3) | 305 (11.5) | 0.528 |
| Hypertension^*^, n (%) | | 265 (19.5) | 527 (19.9) | 0.760 |
| Osteoporosis^‡^, n (%) | | 71 (5.2) | 126 (4.8) | 0.584 |
| Chronic kidney disease^‡^, n (%) | | 333 (24.5) | 668 (25.3) | 0.607 |
| Pneumonia^*^, n (%) | | 40 (2.9) | 40 (1.5) | 0.002 |
| Duration of diabetes^§^ (years) | Median (IQR) | 5.7 (2.8, 9.5) | 5.8 (2.9, 9.6) | 0.482 |
| Duration of medication-treated diabetes^¶^ (years) | No prescription, n(%) | 514 (37.8) | 945 (35.8) | - |
|  | Median (IQR) | 4.8 (2.2, 8.4) | 4.9 (2.2, 8.1) | 0.756 |
| Glucose strip prescriptions (baseline period) | 0 | 1039 (76.4) | 2027 (76.7) | 0.878 |
|  | 1 | 90 (6.6) | 184 (7) |  |
|  | 2–5 | 119 (8.8) | 231 (8.7) |  |
|  | ≥6 | 112 (8.2) | 200 (7.6) |  |
| HbA_1c_ (mmol/mol, IFCC^#^) | Median (IQR) | 51.9 (45.8, 60.7) | 53 (46.4, 61.7) | 0.032 |
| HbA_1c_ (%, DCCT^#^) | Median (IQR) | 6.9 (6.3, 7.7) | 7 (6.4, 7.8) | 0.032 |
| HbA_1c_ (%, DCCT), n (%) | <5.0% | 24 (1.8) | 61 (2.3) | - |
|  | 5.0–5.99% | 149 (11) | 261 (9.9) | - |
|  | 6.0–6.99% | 542 (39.9) | 976 (36.9) | - |
|  | 7.0–7.99% | 380 (27.9) | 791 (29.9) | - |
|  | 8.0–8.99% | 139 (10.2) | 288 (10.9) | - |
|  | 9.0–9.99% | 71 (5.2) | 125 (4.7) | - |
|  | 10.0–10.99% | 29 (2.1) | 84 (3.2) | - |
|  | ≥11.0% | 26 (1.9) | 56 (2.1) | - |
| HbA_1c_ (%, DCCT) within QOF target, ^2^ n (%) | ≤7.5% | 989 (72.7) | 1838 (69.6) | 0.042 |
| Time between HbA_1c_ and index date (days), n (%) | Median (IQR) | 99 (47, 175) | 91 (40, 168) | 0.007 |
| Non-insulin medication,  n (%) | Biguanide or sulphonylurea | 411 (30.2) | 783 (29.6) | - |
|  | Biguanide and sulphonylurea | 193 (14.2) | 385 (14.6) | - |
|  | Other non-insulin | 167 (12.3) | 324 (12.3) | - |
| Non-insulin medication^**^,  n (%) | Biguanide | 716 (52.6) | 1438 (54.4) | 0.300 |
|  | Sulphonylurea | 469 (34.5) | 905 (34.3) | 0.912 |
|  | Alpha glucosidase inhibitor | 4 (0.3) | 9 (0.3) | - |
|  | Prandial glucose regulators | 6 (0.4) | 10 (0.4) | - |
|  | Thiazolidinediones | 140 (10.3) | 291 (11) | 0.521 |
|  | GLP-1 mimetics | 14 (1) | 25 (0.9) | - |
|  | DPP-4 inhibitors | 40 (2.9) | 58 (2.2) | 0.181 |
|  | SGLT2 inhibitors | 0 (0) | 0 (0) | - |

^*^Within 1 year prior to the index date.
^†^Average daily dose within 1 year prior to the index date, calculated as ([count of inhalers × doses in pack]/365) × μg strength.
^‡^Recorded any time prior to the index date.
^§^Estimated as the difference between the date of first diagnosis and the index date.
^¶^Estimated as the difference between the date of first prescription of antidiabetic medication (after a full year of practice data) and the index date.
^#^Last value recorded prior to the index date.
^**^Total per drug class.
COPD = chronic obstructive pulmonary disease; DCCT = Diabetes Control and Complaints Unit; FEV_1_ = forced expiratory volume in 1 second; HbA1c = glycated haemoglobin; ICS = inhaled corticosteroids; IFCC = International Federation of Clinical Chemistry; IQR = interquartile range; LABA = long acting β_2_-agonist; LAMA = long acting muscarinic antagonist; QOF = Quality and Outcomes Framework; SABA = short acting β_2_-agonist; SAMA = short acting muscarinic antagonist.
